# Parameter Trajectory Analysis to Identify Treatment Effects of Pharmacological Interventions

**DOI:** 10.1371/journal.pcbi.1003166

**Published:** 2013-08-01

**Authors:** Christian A. Tiemann, Joep Vanlier, Maaike H. Oosterveer, Albert K. Groen, Peter A. J. Hilbers, Natal A. W. van Riel

**Affiliations:** 1 Department of Biomedical Engineering, Eindhoven University of Technology, Eindhoven, The Netherlands; 2 Netherlands Consortium for Systems Biology, University of Amsterdam, Amsterdam, The Netherlands; 3 Department of Pediatrics, University Groningen, University Medical Center Groningen, Groningen, The Netherlands; 4 Department of Laboratory Medicine, University Groningen, University Medical Center Groningen, Groningen, The Netherlands; Accelrys, United States of America

## Abstract

The field of medical systems biology aims to advance understanding of molecular mechanisms that drive disease progression and to translate this knowledge into therapies to effectively treat diseases. A challenging task is the investigation of long-term effects of a (pharmacological) treatment, to establish its applicability and to identify potential side effects. We present a new modeling approach, called Analysis of Dynamic Adaptations in Parameter Trajectories (ADAPT), to analyze the long-term effects of a pharmacological intervention. A concept of time-dependent evolution of model parameters is introduced to study the dynamics of molecular adaptations. The progression of these adaptations is predicted by identifying necessary dynamic changes in the model parameters to describe the transition between experimental data obtained during different stages of the treatment. The trajectories provide insight in the affected underlying biological systems and identify the molecular events that should be studied in more detail to unravel the mechanistic basis of treatment outcome. Modulating effects caused by interactions with the proteome and transcriptome levels, which are often less well understood, can be captured by the time-dependent descriptions of the parameters. ADAPT was employed to identify metabolic adaptations induced upon pharmacological activation of the liver X receptor (LXR), a potential drug target to treat or prevent atherosclerosis. The trajectories were investigated to study the cascade of adaptations. This provided a counter-intuitive insight concerning the function of scavenger receptor class B1 (SR-B1), a receptor that facilitates the hepatic uptake of cholesterol. Although activation of LXR promotes cholesterol efflux and -excretion, our computational analysis showed that the hepatic capacity to clear cholesterol was reduced upon prolonged treatment. This prediction was confirmed experimentally by immunoblotting measurements of SR-B1 in hepatic membranes. Next to the identification of potential unwanted side effects, we demonstrate how ADAPT can be used to design new target interventions to prevent these.

## Author Summary

A driving ambition of medical systems biology is to advance our understanding of molecular processes that drive the progression of complex diseases such as Type 2 Diabetes and cardiovascular disease. This insight is essential to enable the development of therapies to effectively treat diseases. A challenging task is to investigate the long-term effects of a treatment, in order to establish its applicability and to identify potential side effects. As such, there is a growing need for novel approaches to support this research. Here, we present a new computational approach to identify treatment effects. We make use of a computational model of the biological system. The model is used to describe the experimental data obtained during different stages of the treatment. To incorporate the long-term/progressive adaptations in the system, induced by changes in gene and protein expression, the model is iteratively updated. The approach was employed to identify metabolic adaptations induced by a potential anti-atherosclerotic and anti-diabetic drug target. Our approach identifies the molecular events that should be studied in more detail to establish the mechanistic basis of treatment outcome. New biological insight was obtained concerning the metabolism of cholesterol, which was in turn experimentally validated.

## Introduction

A central aim of medical systems biology is the development of computational models and techniques to study molecular mechanisms that drive disease progression [1]–[13]. One potential contribution of computational modeling is to assess the effectiveness of pharmacological interventions to treat progressive diseases, e.g., Type 2 Diabetes and cardiovascular disease. A complicating factor to simulate and predict the effects of these interventions is the multiscale nature of the affected biological systems. The kinetic computational models in biology are typically constructed to simulate processes at a single timescale, usually capturing short-term dynamics ranging from seconds to hours [14]–[19]. On the other hand, pharmacological interventions usually affect multiple processes that operate at different timescales, which in turn range over an extended time frame. A challenging but particularly relevant task is the investigation of long-term effects of a pharmacological treatment to determine its applicability and to identify potential side effects. Formulating mathematical descriptions of these effects is furthermore complicated by the lack of sufficient information of the underlying network structure and interaction mechanisms. An example is the study of pharmacological treatments associated with metabolic diseases [20], [21]. The acquired experimental data predominantly concern changes in plasma and tissue metabolite concentrations during one or more stages of the treatment. Conversely, it is less well understood to what extent the actual metabolite fluxes change in time and how corresponding processes are modulated by the treatment via interactions with the proteome and transcriptome. As a consequence, in many cases insufficient information is available to explicitly model the interaction mechanisms that modulate the metabolic processes. The lack of mechanistic descriptions of the modulating interactions in a mathematical model, referred to as undermodeling [22], forms a serious complication when studying the effects of a pharmacological treatment by means of computational analyses.

In the present paper we propose a computational approach that overcomes the aforementioned issues. The approach, called Analysis of Dynamic Adaptations in Parameter Trajectories (ADAPT), employs mathematical modeling to predict the long-term effects of a pharmacological intervention. We introduce a concept of time-dependent descriptions of model parameters to study the dynamics of molecular adaptations, making use of experimental data obtained during different stages of an intervention. These model parameters typically represent reaction rate constants (linked to mass action or Michaelis-Menten kinetics), but could be any other quantity expressible in a mathematical model. The progression of adaptations is predicted by identifying necessary dynamic changes in the model parameters to describe the transition between experimental data obtained during different stages of the treatment. The obtained dynamic trajectories of model parameters, as well as metabolite concentrations and -fluxes, are constrained by the network topology and kinetic equations of the molecular processes. As such, our method exploits and integrates the merits from constrained-based modeling approaches (e.g., Flux Balance Analysis) and kinetic modeling. ADAPT is therefore particularly useful to study biological systems from which the network topology is relatively well known, such as the mass fluxes in metabolic pathways. The modulating effects on these pathways via interactions with the proteome and transcriptome, which are less well understood, can be captured by the time-dependent descriptions of the parameters. Hence, as will be shown here, an advantage of ADAPT is that pathway adaptations can be described without the necessity to develop detailed kinetic models of the modulating mechanisms. Moreover, it could provide a means to capture the effects of complex phenomena such as cell differentiation, developmental changes, and aging, that may contribute to the progression of long-term adaptations. The approach originates from our previous work in which computational modeling was used to identify necessary differences in parameters to describe how one phenotype could be evolved from another [1]. While the latter study aimed to explore steady-state differences between two experimentally observed phenotypes, the present study focused on the identification of dynamic adaptations induced by a treatment intervention.

Relevant applications of ADAPT are the investigation of metabolic pathways in relation to progressive diseases such as Type 2 Diabetes and cardiovascular disease. Dyslipidemia is an important risk factor for these diseases, and recognized markers such as plasma triglycerides, LDL- and HDL-cholesterol, are used in clinical settings to assess disease risk and status. However, the underlying molecular mechanisms inducing adaptations in lipid metabolism are not fully understood, complicating the development of effective treatments. In the present study, ADAPT was applied to a model of mouse hepatic lipid and plasma lipoprotein metabolism to identify which metabolic adaptations are induced upon pharmacological treatment of mice by liver X receptor (LXR) agonist T0901317. The family of liver X receptors, LXRα and LXR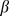, plays a central role in the control of cellular lipid and sterol metabolism. Activation of LXRs by pharmacological agonists promotes the cellular efflux, transport, and excretion of cholesterol from the body, hereby reducing atherosclerotic plaques in rodents [23]. Therefore, LXRs are considered as potential drug targets to treat or prevent atherosclerosis [24]–[26]. However, pharmacological activation of LXR also induces the accumulation of hepatic triglycerides and promotes the secretion of enlarged very-low-density-lipoprotein (VLDL) particles, which complicates the clinical application of LXR agonists [20], [21]. The underlying molecular mechanisms inducing these adaptations in lipid and sterol metabolism are not fully understood. An extensive data set of C57BL/6J mice treated with T0901317 for 0, 1, 2, 4, 7, 14, and 21 days was generated and included in the computational analysis. A remarkable prediction was obtained concerning the scavenger receptor class B1 (SR-B1), a receptor that facilitates the uptake of cholesterol from high-density-lipoproteins (HDL) by the liver. As LXR agonists promote cholesterol efflux from peripheral cells and excretion of cholesterol from the body, it was expected that hepatic SR-B1 expression would be induced upon treatment to accommodate the increased hepatobiliary cholesterol excretion. However, the computational analysis showed that the SR-B1 expression decreased rather than increased upon T0901317 treatment. We recently confirmed this counter-intuitive prediction experimentally by immunoblot analysis of SR-B1 protein expression in hepatic membranes [27]. Results from the computational analysis provided an integrative understanding of the dynamic response induced by T0901317 treatment that was not directly apparent from the experimental data itself. For instance, the results show a clear distinction between the processes that had an early onset and were rapidly activated, and processes that changed progressively during the treatment period.

## Methods

**Figure 1:**
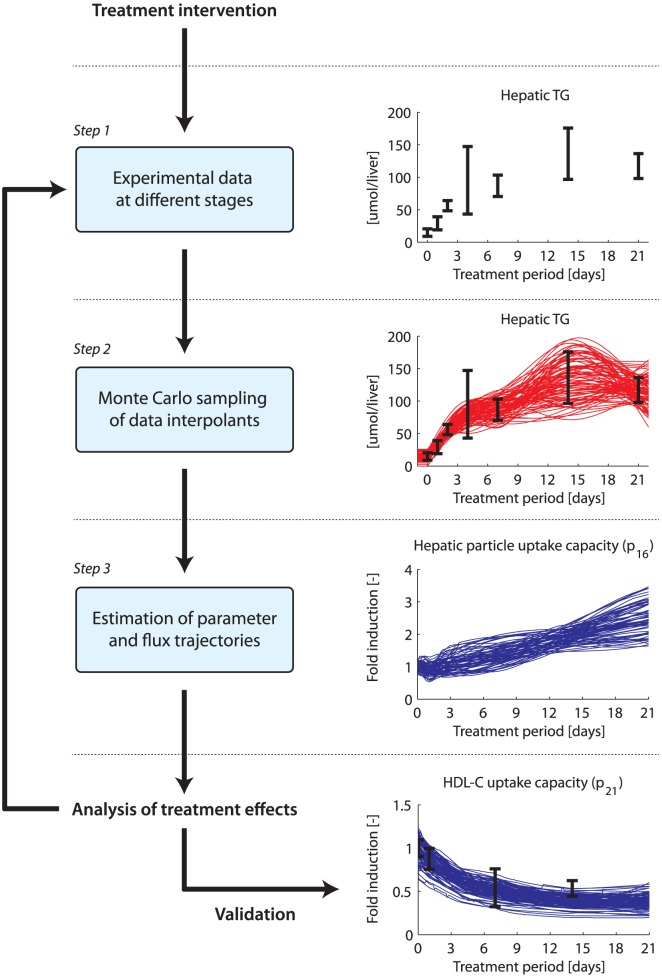
Computational workflow of ADAPT to analyze the effects of a treatment intervention. *Step 1*. Quantitative experimental data was generated at different stages of a treatment intervention. *Step 2*. Cubic smoothing splines were calculated that describe the dynamic trend of the experimental data. To account for experimental and biological uncertainties a collection of splines was calculated using a Monte Carlo approach. *Step 3*. The cubic splines were used as input for the computational approach to iteratively estimate dynamic trajectories of metabolic parameters and fluxes. The additional insights obtained via the computational analysis could be used to design new experiments, and repeat the mentioned steps. For each step an example is given. The data is represented by means 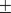 standard deviations.

**Figure 2:**
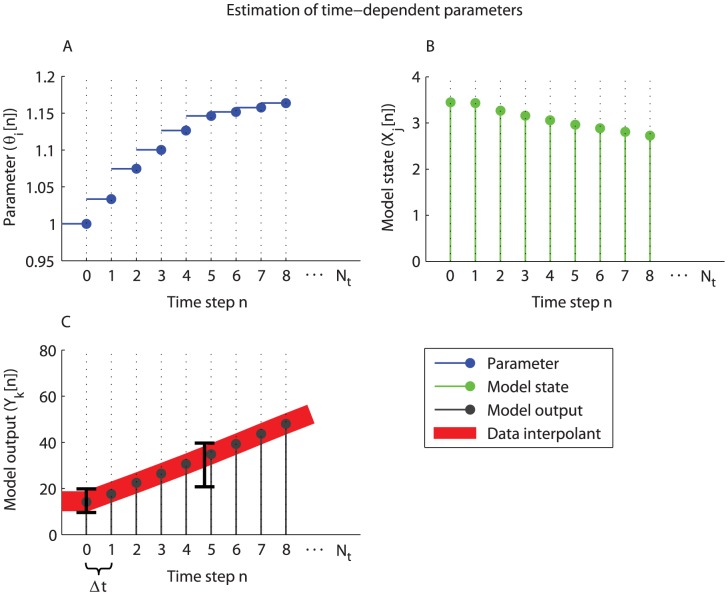
Estimation of time-dependent parameters. The progression of adaptations induced by a treatment intervention is predicted by identifying necessary dynamic changes in the model parameters to describe the transition between experimental data obtained during different stages of the treatment. The time-dependency of the parameters is introduced by dividing a simulation in 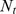 steps of 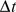 time period. Initially (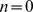) the system is in steady-state and corresponding parameters 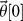 are estimated to describe the experimental data of the untreated phenotype. Subsequently, for each step 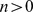 the system is simulated for a time period of 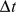 using the final values of the model states of the previous step 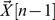 as initial conditions (B). Simultaneously, parameters 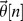 are estimated (A) by minimizing the difference between the data interpolants and corresponding model outputs 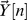 (C). Here, the previously estimated parameter set 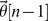 was provided as initial set for the optimization algorithm.

In the following sections we present a step-by-step generic description of the methodology underlying ADAPT. The approach consists of several steps which are discussed below and schematically depicted in Figure 1. A more in-depth illustration of how the time-varying parameters are estimated is provided in Figure 2.

### Ethics statement

A detailed description of the experimental materials and procedures is available (see Supporting Information Text S1). Experimental procedures were approved by the Ethics Committee for Animal Experiments of the University of Groningen.

### Experimental data and Monte Carlo sampling of interpolants

Quantitative experimental data at different stages of a treatment intervention are required to study the dynamics of induced molecular adaptations. In metabolic research the acquired experimental data typically provides information about changes in metabolite concentrations in plasma and tissue compartments [28]–[31] (Figure 1, step 1). In the present study mathematical modeling is employed to generate additional insight on the treatment response by predicting which metabolic parameters and consequently metabolic fluxes necessarily have to change to describe the dynamic trend in the experimental data. The metabolic parameters and fluxes can generally not be considered constant in time. Due to the treatment intervention these quantities typically change in a time-dependent fashion. To allow for estimation of dynamic trajectories of metabolic parameters and fluxes, continuous dynamic descriptions of the experimental data were used as input for ADAPT. For this purpose, cubic smoothing splines were calculated that describe the dynamic trend of the experimental data (Figure 1, step 2). To account for experimental and biological uncertainties a collection of splines was calculated using a Monte Carlo approach. Different random samples of the experimental data were generated assuming Gaussian distributions with means and standard deviations of the data. Subsequently, for each generated sample a cubic smoothing spline was calculated.

### Mathematical modeling to describe the underlying biological system

Fundamental in ADAPT is the development of a computational model that includes mathematical descriptions of the molecular pathways of interest. The present study focused on biological systems that are described by a set of (non)linear ordinary differential equations:
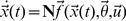
(1)

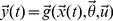
(2)

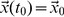
(3)where 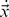 is a vector of first derivatives of molecular species (or states) 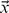 which are given by the topology of the network, matrix 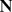, and a set of functions 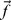. The initial concentrations of 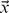 are given by 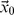. The vector 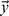 represents the model outputs, which are given by a set of functions 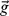 including mathematical expressions that map the model states to specific quantities of interest. Both functions 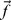 and 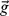 depend on kinetic parameters 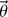 and optional inputs 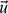.

### Parameterization of the untreated phenotype

In ADAPT the mathematical model is first used to describe the untreated phenotype (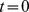). It was assumed that prior to the onset of a treatment intervention the concentrations and fluxes in the biological system were in steady-state. The following protocol was employed to capture multiple parameter sets describing the untreated phenotype. The weighted sum of squared errors between the experimental data of the untreated phenotype and corresponding steady-state model outputs is given by:
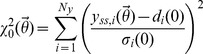
(4)where 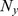 is the number of measurement signals, 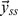 the steady-state model outputs, 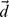 the interpolant functions describing the experimental data and 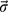 corresponding standard deviations (which are here evaluated at 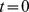). The parameters were estimated by applying a weighted least squares algorithm that minimizes (4):
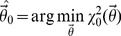
(5)where 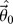 represents the optimized parameter set for the untreated phenotype. A Monte Carlo approach was employed to account for methodological and experimental uncertainties. First, the optimization procedure was repeated for a widely dispersed range of initial parameter values (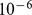 to 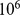). Secondly, in each optimization a different spline function for 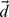 was used. Finally, a collection of 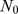 parameter sets is obtained denoted by 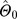 that describe the untreated phenotype:
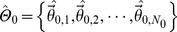
(6)These parameter sets will serve as a starting point from which necessary dynamic changes are identified to describe the transition between experimental data obtained during different stages of the treatment.

### Time-dependent descriptions of model parameters

In many cases insufficient information is available to define the essential interaction mechanisms which are modulated by a specific treatment intervention, let alone to generate explicit mathematical descriptions of these processes. As a consequence, the dynamic adaptations in molecular processes were captured by inferring necessary changes in the model parameters which are therefore time-dependent. Note that it is not known *a priori* how the model parameters change during the experiment. Consequently, it is not possible to perform a dynamic simulation of the entire experiment in one go. This issue was addressed by dividing the simulation of the system in 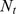 steps of 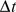 time period using the following discretization (Figure 2):
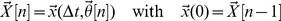
(7)

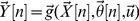
(8)

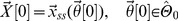
(9)where 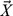 and 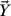 are the discretized quantities of 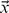 and 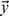 respectively, and 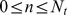 with 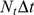 the time period of the entire experiment. The simulation is initiated (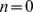) using the steady-state values of the model states 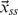 obtained with parameter set 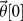, which is part of the collection 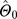 that describes the untreated phenotype. Subsequently, for each step 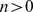 the system is simulated for a time period of 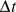 using the final values of the model states of the previous step 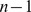 as initial conditions. Note that the model parameters are time-dependent and that each step the system is simulated with a different parameter set. Parameters 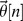 were estimated by minimizing the difference between experimental data (corresponding data interpolant) and corresponding model outputs 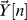. Here, the previously estimated parameter set 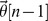 was provided as initial set for the optimization algorithm. It was assumed that the induced adaptations proceed progressively in time. Therefore, highly fluctuating parameter trajectories were considered to be unphysiological. To prevent the occurrence of such behavior, a regularization term, given by the sum of squared derivatives of the normalized parameter values at current step 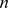, was included in the parameter estimation procedure. An optimized parameter set, denoted by 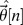, is defined as follows:
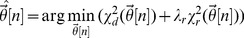
(10)where 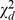 represents the objective function that minimizes the sum of squared differences between the data interpolants and model outputs, and 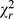 represents the regularization objective function. Equations of the objective functions 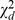 and 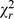 are respectively given by:
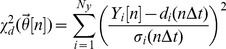
(11)

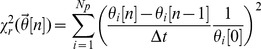
(12)where 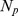 is the number of parameters, and 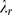 a constant determining the strength of the regularization term. A minimal value for 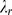 was chosen to bias the data fitting as little as possible [1]. Note that 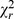 effectuates that changing a parameter is costly, which will therefore be avoided if this is not required to describe the experimental data. Relative derivatives were used to assign equal relevance to all parameters and to avoid domination of the optimization by large absolute values. Finally, trajectories of the parameters (and consequently also for the model states and fluxes) are obtained that describe the transition of the phenotype during the treatment intervention. By repeating the optimization procedure for all initial parameter sets in collection 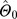 a distribution of trajectories is obtained (Figure 1, step 3).

### Time-dependent sensitivities of trajectories

The calculated trajectories of molecular states, parameters and fluxes can be used for a wide range of analysis techniques to study the induced molecular adaptations. A class of computational techniques that is frequently applied to systems biology models (and complex systems in general) is sensitivity analysis [32]–[35]. One such method is the multi parametric sensitivity analysis (MPSA), which is frequently used to study the relative importance of parameters with respect to model outputs. MPSA is a global sensitivity method that was first proposed in the field of hydrology [36]. More recently, the method was also applied to study biological systems [33], [35], [37], [38]. An advantage of the MPSA method is that it allows to detect combinatorial effects of parameters on model outputs (by varying all parameters simultaneously) that might go unnoticed in local parameter sensitivity analysis based methods (see Supporting Information Text S2 for an example). Here, we briefly discuss the methodology and illustrate how this technique can be applied within the framework of ADAPT. Consider a parameter 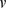 and a model output of interest 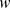. The basic principle of MPSA is to propagate the uncertainty of 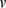 into 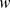 by sampling parameter sets from predefined distributions and evaluate corresponding outcomes of the model output. For the present case these distributions are given by the outcomes of the trajectories corresponding to 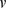 and 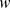 at a specific time step 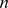. The samples of 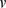 are subsequently classified as acceptable or unacceptable by comparing corresponding outputs 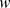 (or some metric involving 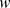) with a threshold. A threshold that is typically used, which was used here as well, is the mean of 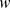 [33], [35], [37], [38]. Next, the samples are sorted according to parameter 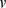 and cumulative distributions of the acceptable (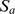) and unacceptable (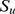) cases are computed:
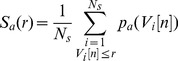
(13)

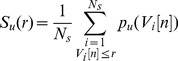
(14)with 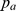 and 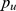 given by:
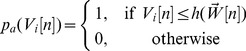
(15)

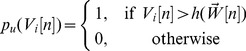
(16)

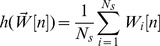
(17)where 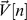 and 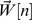 are vectors (sorted according to parameter 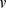) of length 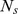 containing the samples of 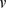 and 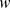 respectively at time step 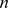. The supremum of the difference between these distributions (Kolmogorov-Smirnov distance) is defined as:
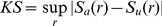
(18)where 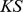 represents the Kolmogorov-Smirnov distance. The 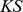 distance serves as a sensitivity metric indicating how strongly acceptance and nonacceptance correlate to parameter 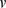, i.e., how sensitive the output 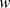 is with respect to the uncertainty in parameter 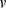. Note that the 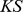 distance is bounded between zero and one, where a higher value indicates a relatively higher importance of the parameter variation to the model output. A critical value for 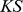 was obtained from the Kolmogorov distribution using a significance level of 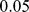 [39], [40]. Another remark is that 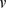 can be any quantity expressed in the mathematical model and is not restricted to parameters. The 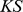 distance was calculated for each time step to study the time-dependency of model sensitivities.

## Results

**Figure 3:**
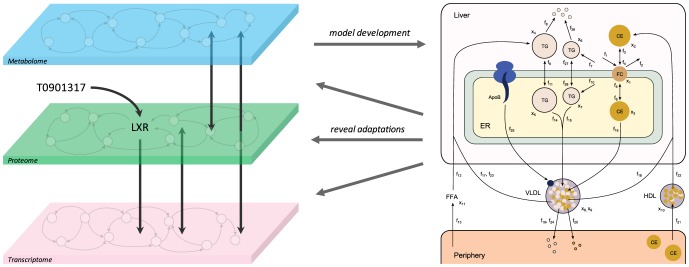
Application of ADAPT to identify adaptations upon pharmacological treatment of mice by LXR agonist T0901317. The intervention starts at the proteome level and subsequently induces adaptations at the other levels (left part, vertical arrows). Mathematical modeling was focused on integrating biological pathways from which the topology is well known and a substantial amount of components were measured quantitatively, i.e. the metabolome level (right part). A detailed description of the mathematical model is presented in Supporting Information Text S3. The modulating effects on metabolic pathways via interactions with the proteome and transcriptome levels are less well understood. At present it is not yet feasible to include a full mechanistic description of these interactions in the model. ADAPT overcomes this problem by introducing time-dependent parameters that incorporate missing modulating effects.

In the present section ADAPT is applied to identify which metabolic adaptations are induced upon pharmacological treatment of mice with LXR agonist T0901317 up to three weeks. The perturbation by means of this treatment starts at the proteome level and subsequently induces adaptations at the other levels (Figure 3, left part). Mathematical modeling was focused on integrating pathways from the metabolome level, as the network topology is relatively well known and the majority of the experimental data was derived from this level (Figure 3, right part). The modulating effects on metabolic pathways via interactions with the proteome and transcriptome levels were captured by time-dependent descriptions of the parameters.

### Pharmacological treatment of LXR using agonist T0901317

An extensive data set of C57BL/6J mice treated with T0901317 for 0, 1, 2, 4, 7, 14, and 21 days was generated and included in the computational analysis. A detailed description of the experimental materials and procedures is available (see Supporting Information Text S1). In brief, the set contains quantitative measures of hepatic triglyceride, free cholesterol, and cholesterylester levels, as well as the fractional contribution of *de novo* lipogenesis to the hepatic triglyceride pool. Furthermore, data on plasma concentrations of triglyceride, total cholesterol, HDL-cholesterol, and free fatty acids (FFA) were included. We also included data on VLDL production, VLDL clearance, VLDL particle size, and VLDL composition. Quantitative data on hepatic cholesterol uptake in untreated mice was derived from [41].

### Computational framework

A mathematical multi-compartment model of mouse hepatic lipid and plasma lipoprotein metabolism was used to predict the dynamics of metabolic adaptations induced upon pharmacological activation of LXR [1]. In the present study several small modifications were made to this model. In brief, the mathematical model contains three compartments representing the liver, blood plasma, and peripheral tissues. The liver includes the production, utilization and storage of triglycerides and cholesterols, as well as the mobilization of these metabolites to the endoplasmic reticulum where they are incorporated into nascent produced VLDL particles. These VLDL particles are subsequently secreted in the plasma compartment and provide nutrients for peripheral tissues. The model furthermore includes the hepatic uptake of free fatty acids from the plasma that predominantly originate from adipose tissue. Finally, the model includes the reverse cholesterol transport pathway, i.e., the net transport of cholesterol from peripheral tissues back to the liver via HDL. A detailed description of the mathematical model, including equations, is available (see Supporting Information Text S3).

The rationale for including the aforementioned biological processes in the mathematical model is to generate a close and balanced match between model complexity and the available experimental data. The level of detail at which certain biological processes can be integrated in a mathematical model is determined by the selection of molecular species, as well as the type and quality of the measurements. Therefore, the model size and complexity of the reaction equations was kept to a minimum. Furthermore, model development was focused on integrating biological pathways from which the topology is well known and a substantial amount of components were measured quantitatively, i.e. mass fluxes at the metabolome level. The network topology of metabolic pathways is relatively well known and is available for different organisms in various pathway databases such as listed in Pathguide (http://www.pathguide.org). The modulating and regulatory effects on metabolic pathways via interactions with the proteome and transcriptome levels are less well understood. At present it is not yet feasible to include a full mechanistic description of these interactions in the model. Note that the computational model does not include any mathematical descriptions of processes involved in LXR activation and its transcriptional response. ADAPT overcomes the problem of undermodeling by introducing time-dependent parameters that account for the missing interactions.

### Analysis of the cascade of induced molecular adaptations

**Figure 4:**
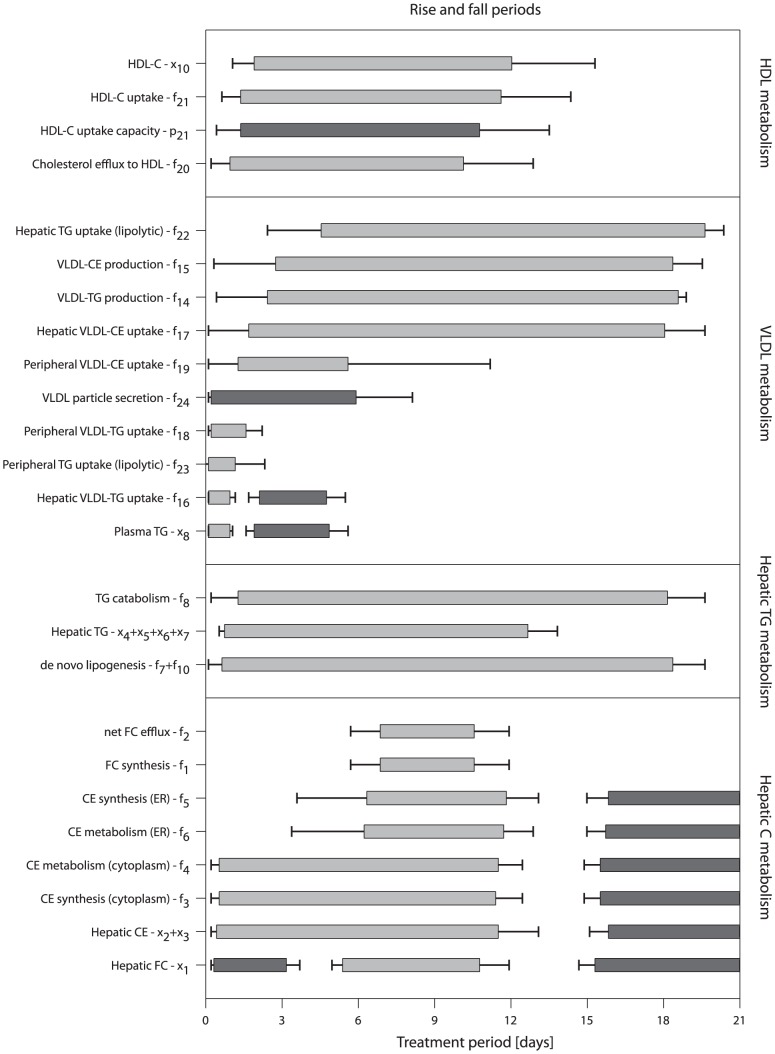
Rise and fall periods of metabolic concentrations, parameters, and fluxes. The rise and fall periods are represented by light-gray and dark-gray bars (median 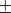 median absolute deviation), respectively. The rise period is defined as the time period during which a trajectory rises from 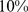 to 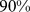 of its maximal value. Similarly, the fall period is defined as the time period during which a trajectory falls from 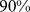 to 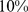 of its maximal value.

The computational workflow of ADAPT was carried out using the computational model and the acquired experimental data. An overview of the experimental data and corresponding spline interpolants that were used as input for ADAPT is presented in Supporting Information Text S4. Parameter trajectories were estimated using 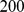 time steps. The impact of changing the number of time steps on the model outputs was investigated (see Supporting Information Text S5). A small value of 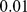 for regularization factor 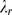 was chosen to bias the data fitting as little as possible (see Supporting Information Text S6). A collection of 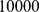 acceptable parameter trajectory sets was obtained describing the experimental data. The dynamic characteristics of the resulting state, parameter, and flux trajectories were investigated to study the cascade of induced molecular adaptations. For this purpose, the rise and fall periods of the trajectories were calculated [42], [43], which provides a broad overview of the response dynamics. The rise period is defined as the time period during which a trajectory rises from 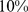 to 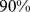 of its maximal value or between two extrema. Similarly, the fall period is defined as the time period during which a trajectory falls from 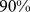 to 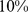 of its maximal value or between two extrema. Figure 4 shows a selection of the rise and fall periods of metabolic concentrations, parameters, and fluxes, clustered by four major metabolic pathways: HDL metabolism, VLDL metabolism, hepatic triglyceride metabolism, and hepatic cholesterol metabolism. The rise and fall periods are respectively represented by light-gray and dark-gray bars (median 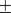 median absolute deviation). A few observations can be made. First, there is a clear distinction between processes that have an early onset and were rapidly activated, and those that changed progressively during the treatment period. This is of importance as the latter processes likely play a crucial role in the long-term effects of the pharmacological intervention. Secondly, the majority of the processes were up-regulated in time. Interestingly, only a small collection of processes included in the model was down-regulated in time compared to the untreated phenotype. Two of these processes, the secretion of VLDL particles and the hepatic HDL-C uptake capacity, were explored in more detail as outlined in the following sections.

### The rate of VLDL particle secretion is reduced upon LXR activation

**Figure 5:**
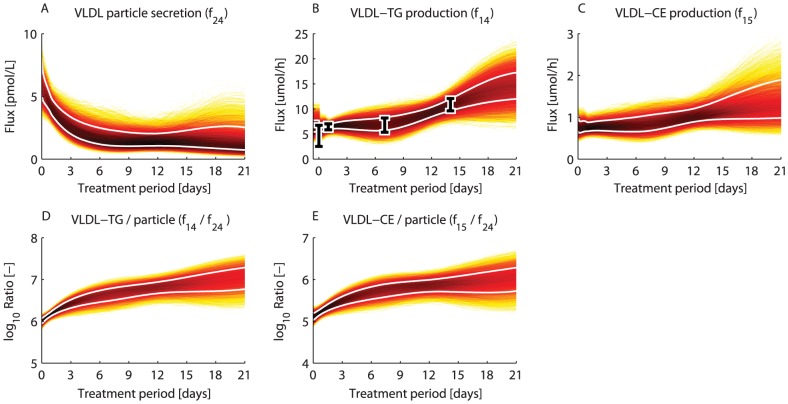
The VLDL particle secretion is reduced upon LXR activation. 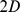
 histograms were calculated from the 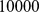 acceptable sets to determine the density of trajectories during the treatment period. A darker color represents a higher density of trajectories in that specific region and time point. The white lines enclose the central 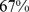 of the densities. A) VLDL particle secretion. B) VLDL-TG production. The data is represented by mean 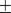 standard deviation. C) VLDL-CE production. D) Ratio of VLDL-TG production to VLDL particle secretion. E) Ratio of VLDL-CE production to VLDL particle secretion.

The estimated trajectories representing the dynamic behavior of the hepatic secretion of VLDL particles to the plasma were investigated. To this end 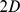 histograms were calculated to determine the density of trajectories during the treatment period (Figure 5). A darker color represents a higher density of trajectories in that specific region and time point. The white lines enclose the central 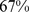 of the densities. It can be observed that the VLDL particle secretion decreased rapidly up to one week of treatment and subsequently stabilized upon prolonged treatment (Figure 5a). Although the secretion of VLDL particles decreased, an increased release of VLDL-TG to the plasma was experimentally observed (Figure 5b). Similarly, the computational analysis showed an increased production of VLDL-CE to the plasma (Figure 5c). According to the model the progressive increase of these fluxes was facilitated by an increased loading of triglycerides and cholesterol onto VLDL particles (Figure 5d,e).

### The hepatic HDL-C uptake capacity is reduced upon LXR activation

**Figure 6:**
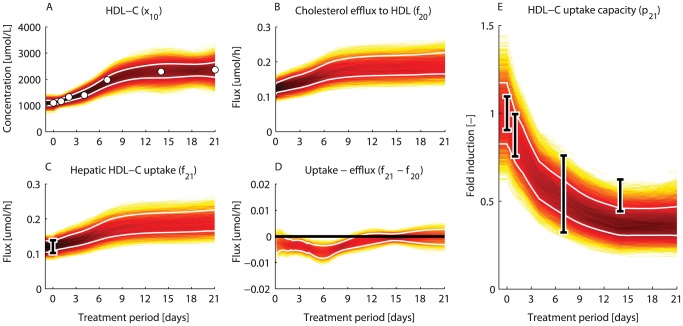
The hepatic HDL-C uptake capacity is reduced upon LXR activation. 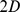
 histograms were calculated from the 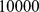 acceptable sets to determine the density of trajectories during the treatment period. A darker color represents a higher density of trajectories in that specific region and time point. The white lines enclose the central 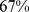 of the densities. A) HDL-C concentration. The white dots represent the experimental data obtained via FPLC measurements from pooled mice plasma. B) Peripheral cholesterol efflux to HDL particles. C) Hepatic uptake of HDL-C. D) Difference between peripheral cholesterol efflux to HDL and HDL-C uptake by the liver. E) Normalized hepatic uptake capacity of HDL-C, which is assumed to be proportional the SR-B1 protein level. This prediction was recently confirmed experimentally by immunoblotting measurements of SR-B1 in hepatic membranes [27] (data represent means 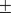 standard deviations). Note that this data serves as an independent validation and was not included in the optimization procedure.

Fast protein liquid chromatography (FPLC) measurements from pooled mice plasma showed an increased level of HDL-C (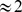 fold increase) after one week of treatment, which remained at this elevated level upon prolonged treatment (Figure 6a). Analysis of the parameter and flux trajectory densities revealed that the rise in HDL-C was initiated by a progressive increment of cholesterol efflux from peripheral cells to HDL particles during the first week of treatment (Figure 6b). This increased efflux was accompanied by an elevated hepatic HDL-C uptake (Figure 6c). Interestingly, only a minor difference between the efflux and uptake rates of HDL-C (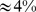) could be observed during the first week of treatment (Figure 6d), implicating that only a small net effect in HDL-C metabolism underlies the marked increase in circulating HDL-C levels (Figure 6a). Although the hepatic uptake of HDL-C was increased, the computational analysis showed that the hepatic HDL-C clearance capacity was reduced upon treatment (Figure 6e). Here, clearance capacity is defined as the ability to clear a certain amount of substrate per time unit from the plasma, which depends on the receptor number and corresponding activity level. The scavenger receptor class B1 (SR-B1) contributes to the hepatic uptake of cholesterol. Recently, we experimentally confirmed that the SR-B1 protein level is reduced in hepatic membranes upon T0901317 treatment (Figure 6e) [27]. Of note, this data serves as an independent validation which was not included in the optimization procedure.

### Analysis and targeting of unwanted side effects

**Figure 7:**
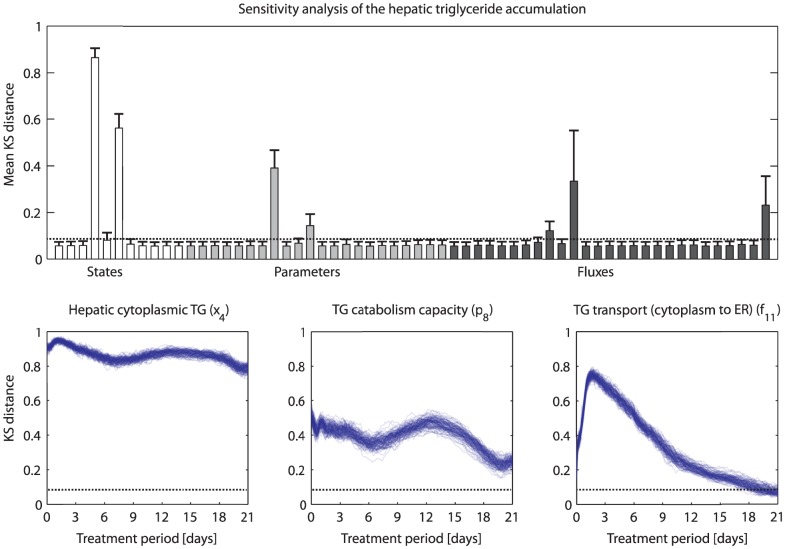
Sensitivity analysis of the hepatic triglyceride accumulation. A sensitivity analysis was performed to identify adapting processes for which the hepatic triglyceride level is sensitive. The quantity of interest 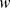 is given by the total hepatic triglyceride pool (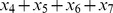). A hundred batches, each containing thousand randomly selected optimized trajectories, were generated. Subsequently, for each batch the temporal sensitivities were calculated. Top) mean 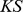 distances for all states, parameters, and fluxes. Bottom) Three examples of dynamic sensitivity profiles. A 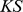 distance was considered significant when it exceeds the critical value indicated by the dotted lines (obtained from the Kolmogorov distribution using a significance level of 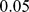).

Besides its beneficial effects on cholesterol metabolism, pharmacological LXR activation also induces unwanted side effects such as the accumulation of triglycerides in the liver. A sensitivity analysis was performed to investigate to which adapting processes the hepatic triglyceride level is sensitive, and therefore potentially played a role in the excessive accumulation of triglycerides in the liver. The quantity of interest 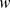 is given by the total hepatic triglyceride pool (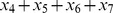). Figure 7 presents the mean 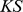 distances for all states, parameters, and fluxes. To assess the consistency of the 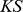 distances, one hundred batches were generated, each containing thousand randomly selected optimized trajectories. Subsequently, the temporal sensitivities were calculated for each batch, providing a measure of the uncertainty associated with the calculated profiles. Changing the number of batches did not qualitatively change the profiles. A 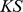 distance was considered significant when it exceeds the critical value indicated by the dotted lines (obtained from the Kolmogorov distribution using a significance level of 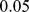). The hepatic triglyceride level was found to be sensitive to adaptations in only a small subset of the model quantities. Three examples of dynamic sensitivity profiles are presented (Figure 7, bottom). With respect to the metabolic states, the total hepatic triglyceride level is sensitive with respect to adaptations in the cytoplasmic triglyceride pool (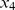 and 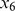) as expected. However, note that the total hepatic triglyceride level is merely negligible sensitive to adaptations in endoplasmic reticulum triglyceride pool (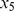 and 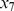). The analysis furthermore shows that the total hepatic triglyceride level is sensitive to changes in the triglyceride catabolism capacity, as well as the transport capacity/fluxes of triglyceride from the cytoplasm to the endoplasmic reticulum. Note that the sensitivity profiles are not static but change during the treatment period, implying that the response of 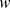 induced by perturbation of 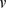 will vary between different stages of the treatment.

**Figure 8:**
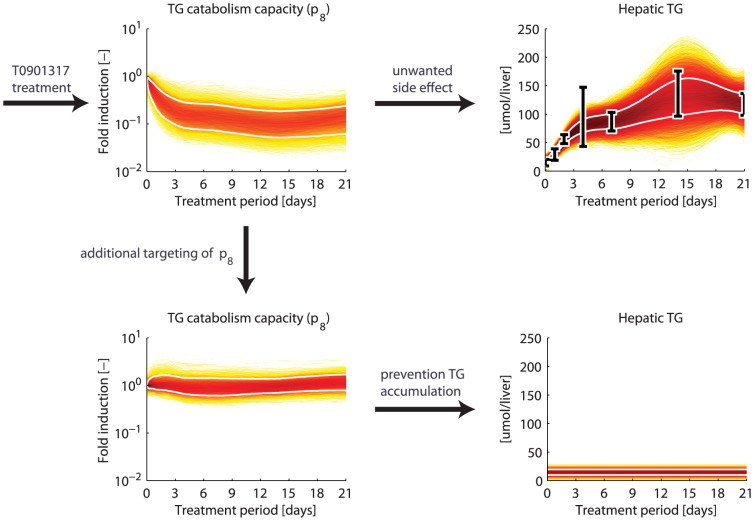
Treatment design to prevent hepatic triglyceride accumulation. A computational analysis was performed to explore the possibility to prevent hepatic triglyceride accumulation upon T0901317 treatment by targeting the triglyceride catabolism capacity (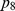). The previous computational analysis showed that the catabolism capacity decreased upon treatment (top left), which is partly responsible for the hepatic triglyceride accumulation (top right). Re-estimation of parameter 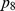, while forcing the total hepatic triglyceride pool to remain constant in time (bottom right), indicates that this objective could be achieved by designing an intervention that maintains the untreated triglyceride catabolism capacity (bottom left). The data is represented by means 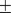 standard deviations. The white lines enclose the central 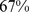 of the densities.

The processes to which the hepatic triglyceride level is sensitive are potential targets for future interventions to prevent the unwanted side effect of excessive triglyceride accumulation in the liver. To illustrate this, we performed a computational analysis to investigate whether it is possible to prevent hepatic triglyceride accumulation upon T0901317 treatment by targeting one of the sensitive quantities, i.e., the triglyceride catabolism capacity (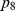). Here, triglyceride catabolism is defined as the hydrolysis of triglyceride into fatty acids and glycerol which are subsequently used in processes such as 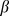-oxidation, gluconeogenesis, ketogenesis, sterol- and phospholipid synthesis. The 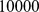 parameter trajectory sets obtained from the previous analysis were used as input to simulate the computational model, with an exception for 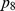. This parameter is iteratively re-estimated (while keeping the other parameters fixed according to their trajectories) to maintain a constant hepatic triglyceride level (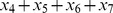) during the treatment intervention. The results of this analysis are depicted in Figure 8. The previous computational analysis showed that the triglyceride catabolism capacity was reduced upon treatment (top left), which is partly responsible for the hepatic triglyceride accumulation (top right). Re-estimation of parameter 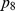, while forcing the total hepatic triglyceride pool to remain constant in time (bottom right), indicates that this objective could be achieved by designing an intervention that maintains the triglyceride catabolism capacity at the level of untreated mice (bottom left). Furthermore, applying this perturbation induced only negligible adaptations in the other metabolite concentrations (see Supporting Information Text S7). Another possibility is to target the triglyceride transport from the cytoplasm to the endoplasmic reticulum (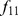 and 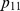). Although targeting 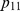 also provides a successful strategy to prevent hepatic triglyceride accumulation, it induces another unwanted side effect, i.e., the accumulation of triglycerides in the plasma (see Supporting Information Text S7).

## Discussion

A relevant topic explored in medical systems biology is the development of computational models and techniques to study the effectiveness of pharmacological interventions to treat progressive diseases. We presented ADAPT, a new modeling approach to analyze the long-term effects of a pharmacological intervention, which is particularly useful to study adaptations in metabolic pathways.

Pharmacological interventions are usually very complex in the sense that they affect multiple processes operating at different levels (metabolome, proteome, and transcriptome) and different timescales. Therefore, in many cases insufficient information is available to define the essential interaction mechanisms which are modulated by a specific treatment intervention. Hence including a full mechanistic description of these interactions in a mathematical model is not possible. ADAPT provides a solution to overcome the problem of undermodeling by introducing time-dependent parameters that account for the missing interactions. We have previously reported a concept of varying parameters [1]. In this study parameter differences/adaptations were estimated to identify molecular differences between phenotypes. The approach was developed to study steady-state differences in concentrations and fluxes between experimentally observed phenotypes. In contrast to ADAPT, no notion of time is integrated in the approach and the dynamics between phenotype transitions were not considered. ADAPT aims to dynamically link different experimentally observed phenotypes (phenotype snapshots) as a function of time, hereby providing an integrated understanding of the outcome of a pharmacological intervention or disease progression.

A concept of time-varying parameters is also used in linear parametric-varying (LPV) control analysis. LPV systems are predominantly applied to design gain-scheduled multivariate controllers [44], [45]. LPV systems are developed for different purposes and there are several essential differences compared to ADAPT. First, LPV controllers are restricted to linear systems. Second, LPV control analysis requires the time variation of parameters to be measured in real-time. The field of Systems Biology deals with the opposite challenge however. In case of biological systems the time variation in parameters is typically not known, and it is therefore the objective of ADAPT to estimate these. Linear time-varying (LTV) systems represent another class of systems in control theory that bear similarities with ADAPT. In LVT systems the input-output characteristics vary with time. These systems are used to design adaptive observers and controllers [46], [47]. These methods also presume that the mechanisms causing the time-dependent differences in output behavior are known or can be measured.

To allow for time-dependent optimization of model parameters according to equation (10) at any time point during a treatment intervention, experimental data at that specific time point is needed. Therefore, continuous dynamic descriptions of the experimental data are required. This issue was addressed by calculating data interpolants. The selection of an appropriate interpolation scheme is important as it determines the dynamic behavior of corresponding model quantities. Considering the uncertainty associated with the acquired experimental data, it was decided to use cubic smoothing splines to describe the experimental data. These descriptions are preferred in cases of noisy observations [48]. Note that the usage of splines provides the possibility to estimate the model parameters in a step-wise manner. However, when considering ‘small’ models (low number of model parameters 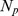) and/or a ‘low’ time resolution (low number of time points 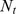), such that the total number of parameters to be estimated (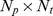) is relatively small, one could opt for an approach to estimate all parameters in a single optimization procedure. This approach is computationally expensive but could provide a means to avoid the usage of data interpolants.

To account for variations in the dynamic behavior as well as experimental and biological uncertainties, a collection of smoothing splines was calculated using a Monte Carlo approach in which random samples of the experimental data were generated. This provides the possibility to determine the propagation of data uncertainty through model predictions, and hence allows to distinguish between predictions that are well constrained and as such can be made confidently, and those that display a large variation in possible outcomes. In case when parameter trajectories (or trajectories of states and fluxes) are not well-constrained by the experimental data, it might be worthwhile to study relative differences of these trajectories compared to the untreated phenotype, which for several cases display consistent behavior (see Supporting Information Text S8). The analysis of parameter and prediction uncertainty is an important topic that triggered the development of various methods [49]–[57]. The sampling of replicates of experimental data and their subsequent utilization in parameter estimation is a common approach to assess prediction uncertainty, a class of methods also referred to as bootstrapping [56], [58]–[63]. Other approaches based on parameter optimization have been proposed to assess the identifiability of parameters [64] and predictions [50] or to probe consistent model behavior (core predictions) among multiple parameter sets [51], [52], [54], [56]. Furthermore, Bayesian methods are available that provide a probabilistic assessment of prediction uncertainty [49], [65]–[67]. A review of the state-of-the-art methods for uncertainty analysis is presented in [68]. An additional analysis was performed to investigate the identifiability of parameters for the untreated phenotype, using ADAPT and the Profile Likelihood method [64] (see Supporting Information Text S9).

ADAPT was applied to a model of hepatic lipid and plasma lipoprotein metabolism to predict the metabolic adaptations induced upon pharmacological treatment of mice with the LXR agonist T0901317. As values for model parameters need to be inferred from experimental data, mathematical modeling was focused on integrating biological pathways from which a substantial amount of components were measured quantitatively. For the present case these predominantly concerned measurements of metabolite concentrations in the liver and plasma. Therefore, mathematical modeling was centered on integrating corresponding pathways at the metabolic level. Interactions and processes at the proteome and transcriptome levels were not included, as insufficient information of the underlying network structure and interaction mechanisms was available. Conversely, these modulating effects were captured by inferring necessary changes in the model parameters. Note that the computational model does not include any mathematical descriptions of processes involved in LXR activation and its transcriptional response. We were able to quantitatively integrate data of untreated mice, as well as mice treated with T0901317 up to three weeks into a consistent model. The presented model predictions are in good agreement with experimental observations and our previous results [1]. An additional analysis was performed that confirms that the parameters have to change in a time-dependent manner to describe the experimental data. It was not possible to describe the experimental data by simulating the system with time-constant parameters or a step-wise response in the parameters (see Supporting Information Text S10). The calculated trajectories of metabolic states, parameters and fluxes can be used for a wide range of analysis techniques to study the induced molecular adaptations. Several of its potential applications were presented here, e.g., sensitivity analysis, and therapy design.

The obtained trajectories provided the opportunity to study the cascade of metabolic adaptations. Our results show a clear distinction between processes that had an early onset and were rapidly activated, and processes that changed progressively during the treatment period. For instance, the peripheral uptake of triglycerides via lipolytic enzymes is rapidly induced, while this process is induced progressively in the liver (Figure 4, 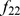 vs. 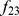). Analysis of the trajectories revealed that the majority of the processes were up-regulated in time. Interestingly, only a small subset of the included processes were down-regulated in time. One example concerns the secretion of VLDL particles. The computational analysis revealed that the secretion decreased rapidly up to one week of treatment and subsequently stabilized upon prolonged treatment (Figure 5a). This model prediction is consistent with the following experimental observations. A reduced level of hepatic apolipoprotein B mRNA (each VLDL particle contains one apolipoprotein B protein) was observed in T0901317 treated mice [20], [21], [69]. Although the secretion of VLDL particles decreased upon T0901317 treatment, the VLDL-mediated transport of triglycerides and cholesterol to the plasma increased progressively (Figure 5b,c). This was accomplished by an increased loading of these lipids onto VLDL particles (Figure 5d,e), resulting in an enlargement of the particle volume (see Supporting Information Text S4).

Model predictions furthermore indicated that the efflux of cholesterol from peripheral tissues to HDL particles increased up to one week of treatment (Figure 6b), which most likely resulted from the induction of the cholesterol transporters ABCA1 and ABCG1 in peripheral tissues. The increased cholesterol efflux was closely followed by an increased uptake of HDL-C by the liver (Figure 6c). However, a minor difference between the efflux and uptake rates of HDL-C can be observed during the first week of treatment (Figure 6d), resulting in an elevated plasma HDL-C level (Figure 6a). Another interesting prediction obtained from the computational analysis concerns SR-B1, a receptor that facilitates the uptake of cholesterol from HDL by the liver. As LXR agonists promote the efflux of cholesterol from the periphery and excretion of cholesterol from the body, it was expected that SR-B1 expression level would be induced upon treatment to accommodate the increased hepatobiliary flux. In contrast, the computational analysis showed that the hepatic capacity to clear HDL-C (assumed to be proportional to the SR-B1 level) was reduced upon treatment (Figure 6e). This counter-intuitive prediction was recently confirmed experimentally by immunoblotting analysis of SR-B1 protein expression in hepatic membranes [27]. Hence, the increased HDL-C concentration is not only a consequence of increased peripheral cholesterol efflux to HDL particles [20], [70] but also of impaired SR-B1-mediated cholesterol uptake by the liver.

The calculated trajectories form a hypothesis on how the various metabolic states, parameters, and fluxes changed during the treatment intervention. These trajectories can subsequently be exploited to establish the efficacy of a treatment and to identify its potential side effects. In case unwanted side effects occur, the trajectories may be used to design new or additional target interventions to prevent these. Here, we presented an example concerning the excessive accumulation of triglycerides in the liver upon LXR activation. First, a sensitivity analysis was performed to identify adapting processes to which the hepatic triglyceride level is sensitive, and therefore potentially contributed to the accumulation of triglycerides in the liver (Figure 7). Subsequently, we performed a computational analysis to investigate the possibility to maintain normal hepatic triglyceride levels upon T0901317 treatment by targeting the triglyceride catabolism capacity (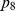), one of the sensitive quantities. This parameter is iteratively re-estimated to maintain a constant hepatic triglyceride content during the treatment intervention, while keeping the other parameters fixed according to their trajectories obtained from the original analysis (Figure 8). Note that although all parameters are fixed (with an exception for 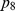), the concentrations and fluxes can change due to the targeting of 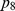. Here, we assumed that the targeting of 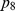 induces merely negligible adaptations on the model parameters. Figure 8 shows to what extent the catabolism capacity should be targeted in order to maintain a hepatic triglyceride content within the normal range. Such information can subsequently be used to design specific target interventions to achieve this. One option could be to increase mitochondrial fatty acid oxidation, thereby increasing triglyceride catabolism. Several therapeutic strategies to achieve this have been proposed in recent years [71]–[73]. Another strategy would be to inhibit Acetyl-CoA carboxylase (ACC), the enzyme that catalyzes malonyl-CoA synthesis. malonyl-CoA acts as an allosteric inhibitor of mitochondrial fatty acid oxidation. ACC inhibition will therefore reduce malonyl-CoA levels, hence releasing the inhibitory effect on fatty acid catabolism [74], [75].

In conclusion, we presented ADAPT, a new modeling approach to evaluate the consequences of a pharmacological intervention. The calculated trajectories of metabolic states, parameters and fluxes can be used for a wide range of analytical techniques to study the molecular adaptations. They provide insight in the affected underlying biological systems and identify the molecular events that should be studied in more detail to unravel the mechanistic basis of treatment outcome.

## Supporting Information

Text S1 Description of the experimental procedures.
(PDF)

Text S2 An example of the multi parametric sensitivity analysis (MPSA) method is demonstrated and compared with a local sensitivity analysis method.
(PDF)

Text S3 Description of the mathematical model of hepatic lipid and plasma lipoprotein metabolism, including an overview of the states, parameters, fluxes, and ordinary differential equations.
(PDF)

Text S4 An overview of the experimental data and corresponding spline interpolants that were used as input for ADAPT.
(PDF)

Text S5 Analysis of the influence of changing the number of time steps used in ADAPT on the model outputs and regularization error.
(PDF)

Text S6 Analysis of the influence of changing the regularization strength on the model outputs and regularization error.
(PDF)

Text S7Analysis of unwanted side effects when targeting 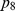 or 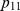 to prevent the excessive accumulation of triglycerides in the liver upon T0901317 treatment.(PDF)

Text S8 Analysis of relative adaptations of trajectories to identify consistent model behavior.
(PDF)

Text S9 Analysis of the identifiability of parameters for the untreated phenotype, using ADAPT and the Profile Likelihood method.
(PDF)

Text S10 Comparison of model outputs obtained with time-dependent parameters used in ADAPT and an analysis using time-constant parameters.
(PDF)
