# Supplementary material for: Parameter Trajectory Analysis to Identify Treatment Effects of Pharmacological Interventions
Source: PLoS Comput Biol. 2013 Aug 1;9(8):e1003166. doi: 10.1371/journal.pcbi.1003166 (PMC3731221; doi:10.1371/journal.pcbi.1003166)
Supplement: Text S1 — Description of the experimental procedures. (PDF) [file pcbi.1003166.s001.pdf]

Parameter Trajectory Analysis to Identify Treatment Effects of Pharmacological Interventions (Supporting Information Text S1)

C.A. Tiemann, J. Vanlier, M.H. Oosterveer, A.K. Groen, P.A.J. Hilbers, N.A.W. van Riel

Experimental procedures

Animals and experimental design

Male C57Bl/6J mice (Charles River, L'Arbresle Cedex, France) were housed in a light- and temperature-controlled facility (lights on 6:30 AM-6:30 PM, 21 °C) and fed a standard laboratory chow diet (RMH-B, Abdiets, Woerden, The Netherlands) containing T0901317 (0.015% wt/wt; ∼ 50 mg/kg) for 1, 2, 4, 7, 14, or 21 days. Untreated controls received non-supplemented laboratory chow. After 1 day, 7 days, and prior to sacrifice on day 14, a small blood sample was taken from 4-h fasted (8-12 AM) mice by tail bleeding to evaluate plasma lipoprotein profiles. All animals had free access to drinking water. During the final 24-hours of the treatment period, the different groups of mice received sodium [1-^{13}C]-acetate (99 atom %, Isotec/Sigma-Aldrich, St. Louis, MO) via the drinking water (2%). On the last treatment day, 4-h fasted (8-12 AM) animals were sacrificed by cardiac puncture under isoflurane anaesthesia. Livers were quickly removed, freeze-clamped and stored at -80 °C. Blood was centrifuged (4000xg for 10 minutes at 4 °C) and plasma was stored at -20 °C. Experimental procedures were approved by the Ethics Committee for Animal Experiments of the University of Groningen.

Liver and plasma metabolites and plasma lipoprotein analysis

Plasma lipoproteins were separated by fast protein liquid chromatography (FPLC) gel filtration using a superose 6 column (GE Healthcare, Uppsala, Sweden) [1]. Triglyceride contents of the collected FPLC fractions were determined using a commercially available kit (Roche Diagnostics, Mannheim, Germany). Plasma non-esterified fatty acid profiles were analyzed as previously described [2]. Frozen liver was homogenized in ice-cold PBS. Hepatic triglyceride and total cholesterol contents were assessed using commercial available kits (Roche Diagnostics) after lipid extraction [3]. Hepatic triglyceride fractions were obtained from lipid extracts using Isolute SPE NH_2 columns (Biotage AB, Uppsala, Sweden) [4, 5].

Fractional contribution of de novo lipogenesis

Hepatic triglyceride fractions obtained by lipid extraction were hydrolyzed, and the free fatty acids were extracted and derivatized [6]. The fatty acid-mass isotopomer distributions were determined by GC-MS and used in mass isotopomer distribution analysis (MIDA) to calculate fractional palmitate and oleate synthesis rates from de novo lipogenesis [6].

Quantification of VLDL-TG production rates

Separate groups of mice were injected intravenously with Triton WR1339 (0.5 g/kg body weight) as a 125 mg/mL solution in PBS after a 4-hour fast (8-12 AM). Blood samples were drawn by retro-orbital bleeding into heparinized tubes at 0, 30, 60, 120, and 240 min after injection. After the last blood draw, animals were sacrificed by cardiac puncture under isoflurane anaesthesia. Blood was centrifuged (10 minutes, 4000xg) to obtain plasma. Plasma triglyceride levels and triglyceride production rates were determined as described [7]. Nascent VLDL ($d< 1.006$) was isolated from the final plasma sample of each animal using a Optima TM LX tabletop ultracentrifuge (Beckman Instruments Inc., Palo Alto, CA) at 108,000 rpm for 150 minutes.

Determination of nascent VLDL composition and particle size

Triglyceride, cholesterol and phospholipid concentrations of the nascent VLDL particles were determined using commercially available kits (Roche Diagnostics and Wako Chemicals). Protein concentrations were determined using the BCA Kit (Pierce, Rockford, IL). VLDL particle diameter D was estimated according to [8] using the following formula: $D = 60 \cdot ((0.211 \cdot TG/PL) + 0.27))$, where D is given in [nm].

Quantification of VLDL catabolic rates

VLDL was isolated from pooled plasma of fasting healthy human subjects by ultracentrifugation ($d< 1.006$). VLDL was iodinated using the iodine monochloride method [9]. Free iodine was removed by passing over a PD-10 column (GE Healthcare, Diegem, Belgium) followed by extensive dialysis against PBS. More than 95% of the VLDL radioactivity was precipitable by trichloroacetic acid, and less than 6% of the radioactivity was associated with the lipid fraction of VLDL. Separate groups of mice were injected with 0.5 Ci of 125I-VLDL via the tail vein after a 4-hour fast (8-12 AM). Blood samples were taken by retro-orbital bleeding after 0, 15, 30, 60, 120 and 240 minutes and plasma radioactivity was determined using a Cobra II γ counter (Packard Instruments, Downers Grove, IL). Plasma decay curves for the tracer were generated by dividing plasma radioactivity at each time point by the radioactivity present at the initial 1min time point. Fractional catabolic rates were calculated from the area under the plasma disappearance curves fitted to a bicompartmental model using SAAM-II (version 1.2.1; SAAM Institute, University of Washington, Seattle, WA) [1].

Immunoblotting procedures

Protein concentrations in liver homogenates containing protease inhibitors (Complete; Roche Diagnostics) were determined using the BCA Kit (Pierce). Volumes of VLDL containing equal amounts of triglyceride were pooled and lipids were extracted with methanol and cold ether. The remaining VLDL proteins were subjected to SDS-PAGE. Apolipoprotein B100 and apolipoprotein B48 were determined using antibodies against antimouse apoB raised in rabbit (Biodesign, Saco, ME). Horseradish peroxidase-conjugated antirabbit antibodies from donkey (Amersham Pharmacia Bioscience, GE Healthcare) was used as a secondary antibody for all immunoblots. Protein bands were detected using SuperSignal West Pico Chemiluminescent Substrate System (Pierce). Band-densities were determined by using a Gel Doc XR system (Biorad, Hercules CA, USA).

References

1. Tietge U, Maugeais C, Cain W, Grass D, Glick J, et al. (2000) Overexpression of secretory phospholipase a2 causes rapid catabolism and altered tissue uptake of high density lipoprotein cholesteryl ester and apolipoprotein ai. Journal of Biological Chemistry 275: 10077–10084.

2. Duran M, Bruinvis L, Ketting D, de Klerk J, Wadman S (1988) Cis-4-decenoic acid in plasma: a characteristic metabolite in medium-chain acyl-coa dehydrogenase deficiency. Clinical chemistry 34: 548–551.

3. Bligh E, Dyer W (1959) A rapid method of total lipid extraction and purification. Canadian journal of biochemistry and physiology 37: 911–917.

4. Kaluzny M, Duncan L, Merritt M, Epps D (1985) Rapid separation of lipid classes in high yield and purity using bonded phase columns. Journal of Lipid Research 26: 135–140.

5. Hoving E, Jansen G, Volmer M, Van Doormaal J, Muskiet F (1988) Profiling of plasma cholesterol ester and triglyceride fatty acids as their methyl esters by capillary gas chromatography, preceded by a rapid aminopropyl-silica column chromatographic separation of lipid classes. Journal of Chromatography B: Biomedical Sciences and Applications 434: 395–409.

6. Oosterveer M, Van Dijk T, Tietge U, Boer T, Havinga R, et al. (2009) High fat feeding induces hepatic fatty acid elongation in mice. PLoS One 4: e6066.

7. Gautier T, Tietge U, Boverhof R, Perton F, Le Guern N, et al. (2007) Hepatic lipid accumulation in apolipoprotein ci-deficient mice is potentiated by cholesteryl ester transfer protein. Journal of lipid research 48: 30–40.

8. Fraser R (1970) Size and lipid composition of chylomicrons of different svedberg units of flotation. Journal of lipid research 11: 60–65.

9. Fidge N, Poulis P (1974) Studies on the radioiodination of very low density lipoprotein obtained from different mammalian species. Clinica Chimica Acta 52: 15–26.
