# Supplementary material for: Parameter Trajectory Analysis to Identify Treatment Effects of Pharmacological Interventions
Source: PLoS Comput Biol. 2013 Aug 1;9(8):e1003166. doi: 10.1371/journal.pcbi.1003166 (PMC3731221; doi:10.1371/journal.pcbi.1003166)
Supplement: Text S2 — An example of the multi parametric sensitivity analysis (MPSA) method is demonstrated and compared with a local sensitivity analysis method. (PDF) [file pcbi.1003166.s002.pdf]

Parameter Trajectory Analysis to Identify Treatment Effects of Pharmacological Interventions (Supporting Information Text S2)

C.A. Tiemann, J. Vanlier, M.H. Oosterveer, A.K. Groen, P.A.J. Hilbers, N.A.W. van Riel

Multi parametric sensitivity analysis

The multi parametric sensitivity analysis (MPSA) method is demonstrated, and compared with a local sensitivity measure, by applying it to a simple toy model. Consider the following mathematical model of the complex formation of species A and B to AB (Figure S1):

$$\frac{d[A]}{dt} = k_1 - k_3[A][B]$$
$$\frac{d[B]}{dt} = k_2 - k_3[A][B]$$
$$\frac{d[AB]}{dt} = k_3[A][B] - k_4[AB]$$

A reference simulation (Figure S1) is obtained using values for the initial conditions and parameters as presented in Table S1. The output of interest y is given by the steady-state value of $[AB]$. A local parametric sensitivity analysis (LPSA) was performed by calculating the following measure for each parameter k_i [1]:

$$S_i = \frac{dy/y_{ref}}{dk_i/k_{i_{ref}}}$$

where dy represents the difference in output compared to the reference output y_{ref} upon increasing the reference parameter value $k_{i_{ref}}$ by dk_i. The parameters were individually increased by 50% with respect to the reference values. The results are depicted in Figure S2 (top left). The output is only sensitive to changes in parameter k_4. Subsequently, the MPSA method was employed to determine the relative importance of the parameters with respect to output of interest. A collection of $N_s = 10000$ randomly sampled parameter sets was generated, using uniform distributions between $\pm 50\%$ of the reference parameter values. Note that all parameters are varied simultaneously. For each generated parameter set the model is simulated and the output of interest is calculated. Next, for each parameter individually the following procedure was performed. The samples are sorted according to the selected parameter and cumulative distributions of the acceptable (S_a) and unacceptable (S_u) cases are computed:

$$S_a(r) = \frac{1}{N_s} \sum_{\substack{j=1 \\ k_{i,j} \leq r}}^{N_s} p_a(k_{i,j}) \tag{1}$$

$$S_u(r) = \frac{1}{N_s} \sum_{\substack{j=1 \\ k_{i,j} \leq r}}^{N_s} p_u(k_{i,j}) \tag{2}$$

Table S1. Values for the initial conditions and parameters used in the toy model

State / Parameter	Value
$[A](t_0)$	1
$[B](t_0)$	2
$[AB](t_0)$	0
k_1	1
k_2	1
k_3	1
k_4	1

with p_a and p_u given by:

$$p_a(k_{i,j}) = \begin{cases} 1, & \text{if } k_{i,j} \leq h(\vec{y}) \\ 0, & \text{otherwise} \end{cases} \tag{3}$$

$$p_u(k_{i,j}) = \begin{cases} 1, & \text{if } k_{i,j} > h(\vec{y}) \\ 0, & \text{otherwise} \end{cases} \tag{4}$$

$$h(\vec{y}) = \frac{1}{N_s} \sum_{i=1}^{N_s} y_{i,j} \tag{5}$$

where $\vec{y}$ is a vector of length N_s containing the model outputs corresponding to all sampled parameter sets. Furthermore, $k_{i,j}$ and $y_{i,j}$ respectively denote the ith parameter and ith model output from the jth sampled parameter set. The supremum of the difference between these distributions (Kolmogorov-Smirnov distance) is given by:

$$KS = \sup_r \left| S_a(r) - S_u(r) \right| \tag{6}$$

where KS represents the Kolmogorov-Smirnov distance. The KS distances are presented in Figure S2 (top right). For each parameter corresponding cumulative distributions and KS distance are depicted (bottom plots). A critical value for the KS distance (indicated by the dashed line) was obtained from the Kolmogorov distribution using a significance level of 0.05 [2,3]. A few observations can be made. Results from both the LPSA and MPSA indicate that the model output is most sensitive to changes in parameter k_4. This is expected because k_4 directly acts on the model output. Furthermore, both analyses indicate that the model output is not sensitive for k_3. This is also expected as k_3 will only influence the dynamics, but not the steady-state of $[AB]$. However, different results are obtained concerning parameters k_1 and k_2. The LPSA method attributes no relative importance to these parameters. Indeed, when these parameters are varied individually they will only induce modest changes to the steady-state value of $[AB]$. However, when both parameters are changed simultaneously this could have an impact on the steady-state value of $[AB]$. Hence, depending on the situation the model output could display sensitivity with respect to changes in these parameters. An advantage of the MPSA method is that it allows to detect these combinatorial effects of parameters on model outputs that might go unnoticed in LPSA based methods.

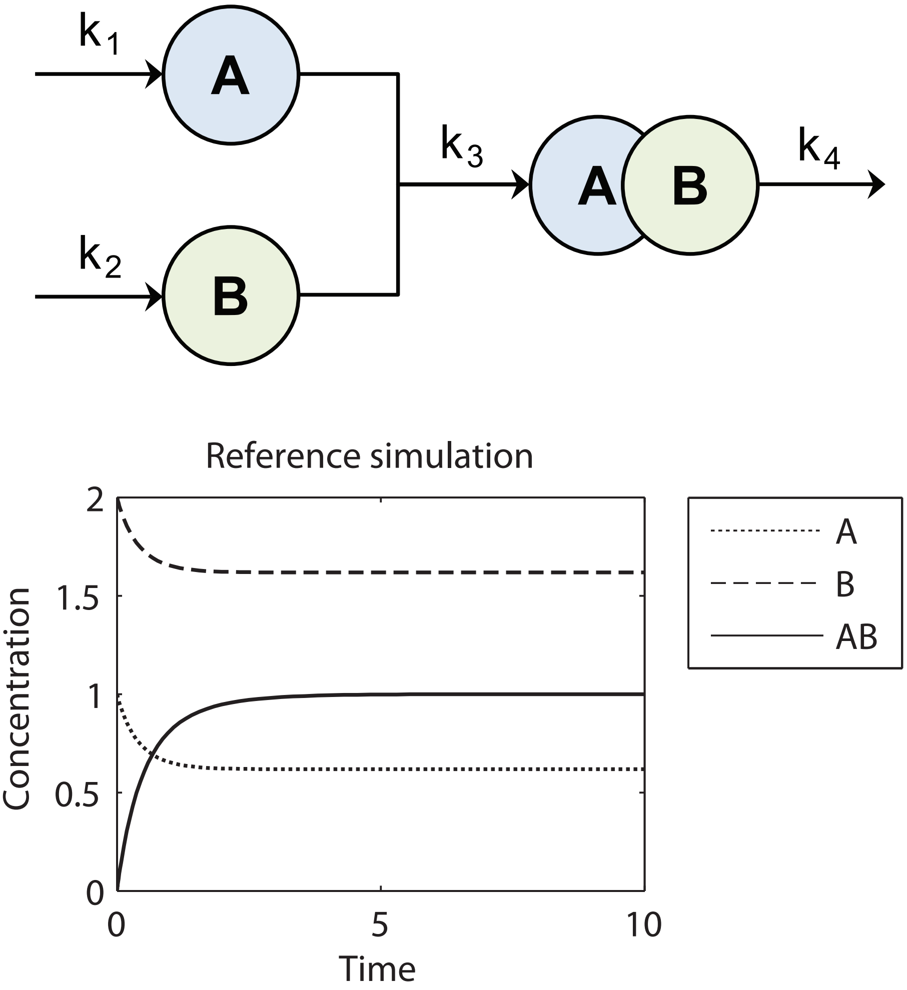

Figure S1. Overview of the toy model. The MPSA method is demonstrated by applying it to a simple model of complex formation of species A and B to AB. Top) schematic overview of the model. Bottom) reference simulation obtained with values for the initial conditions and parameters as presented in Table S1.

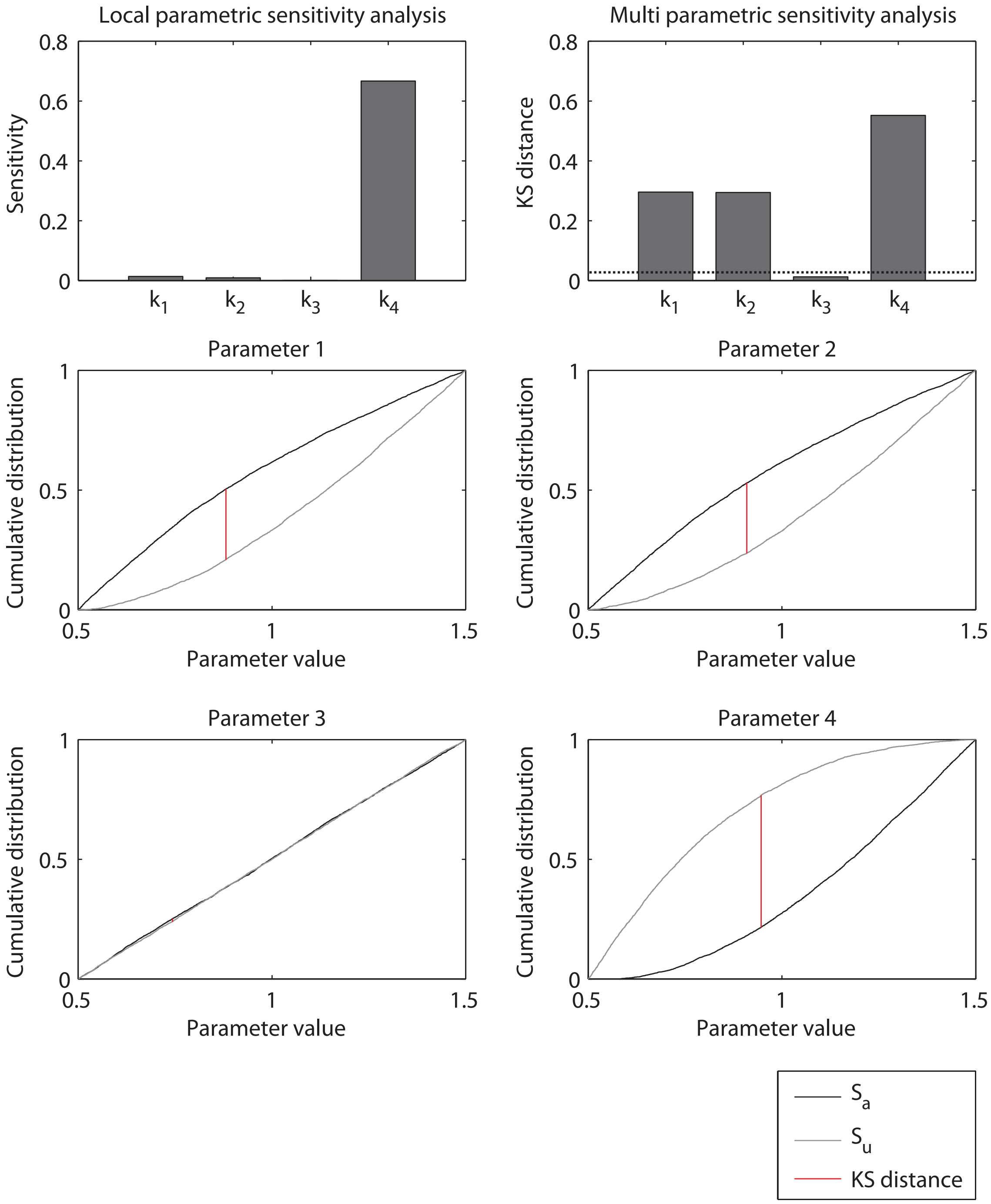

Figure S2. Parametric sensitivity analysis. Top left) Results obtained with the LPSA method. Top right) KS distances obtained with the MPSA method. A significant KS distance is indicated by the dashed line. Bottom plots) For each parameter corresponding cumulative distributions and KS distance are depicted.

References

1. van Riel N (2006) Dynamic modelling and analysis of biochemical networks: mechanism-based models and model-based experiments. Briefings in Bioinformatics 7: 364–374.

2. Wang J, Tsang WW, Marsaglia G (2003) Evaluating kolmogorov's distribution. Journal of Statistical Software 8.

3. Pearson E, Hartley H (1958) Biometrika tables for statisticians. Cambridge: Cambridge University Press, 240 pp.
