# Supplementary material for: Parameter Trajectory Analysis to Identify Treatment Effects of Pharmacological Interventions
Source: PLoS Comput Biol. 2013 Aug 1;9(8):e1003166. doi: 10.1371/journal.pcbi.1003166 (PMC3731221; doi:10.1371/journal.pcbi.1003166)
Supplement: Text S3 — Description of the mathematical model of hepatic lipid and plasma lipoprotein metabolism, including an overview of the states, parameters, fluxes, and ordinary differential equations. (PDF) [file pcbi.1003166.s003.pdf]

Parameter Trajectory Analysis to Identify Treatment Effects of Pharmacological Interventions (Supporting Information Text S3)

C.A. Tiemann, J. Vanlier, M.H. Oosterveer, A.K. Groen, P.A.J. Hilbers, N.A.W. van Riel

Computational model

Description

A mathematical multi-compartment model of mouse hepatic lipid and plasma lipoprotein metabolism was used to predict the dynamics of metabolic adaptations induced upon pharmacological activation of LXR [1]. The mathematical model contains three compartments representing the liver, blood plasma, and periphery (Figure S3). The liver includes the production, utilization and storage of triglycerides and cholesterol, as well as the mobilization of these metabolites to the endoplasmic reticulum where they are incorporated into nascent VLDL particles. These VLDL particles are subsequently secreted in the plasma compartment and provide nutrients for peripheral tissues. The model furthermore includes the hepatic uptake of free fatty acids from the plasma that predominantly originate from adipose tissue. Finally, the model includes the reverse cholesterol transport pathway, i.e., the net transport of cholesterol from peripheral tissues back to the liver via HDL. In the present study several small modifications were made compared to the model used in [1]. As FFA derived from triglycerides are oxidized in mitochondria which are located in the cytoplasm, it was assumed that no oxidation takes place in the endoplasmic reticulum compartment [2]. Therefore, latter oxidation reaction was discarded from the mathematical model. Furthermore, the FFA concentration in the plasma was explicitly modeled using an ordinary differential equation ($\frac{d[x_{FFA}]}{dt}$), which includes a net additive efflux of FFA from adipose tissue and a subtractive uptake flux of FFA by the liver. Finally, two states were added to the model ($x_{TGdnl_{cyt}}$ and $x_{TGdnl_{ER}}$) representing the fractions of *de novo* produced triglycerides in the cytoplasm and endoplasmic reticulum. This adjustment was required to allow for relating experimental data of the fractional contribution of *de novo* lipogenesis to the model.

Model equations

The mathematical model contains eleven metabolic species $\vec{x}$ (Table S2) interlinked by twenty-nine flux interactions (Table S3). The flux equations are based on mass-action kinetics. The ordinary differential

Table S2. Overview and description of the state variables included in the mathematical model.

State	Name	Description
x_1	x_{FC}	Hepatic free cholesterol
x_2	$x_{CE_{cyt}}$	Hepatic cholesteryl ester (cytosol)
x_3	$x_{CE_{ER}}$	Hepatic cholesteryl ester (ER)
x_4	$x_{TG_{cyt}}$	Hepatic triglyceride (cytosol)
x_5	$x_{TG_{ER}}$	Hepatic triglyceride (ER)
x_6	$x_{TGdnl_{cyt}}$	Hepatic *de novo* triglyceride (cytosol)
x_7	$x_{TGdnl_{ER}}$	Hepatic *de novo* triglyceride (ER)
x_8	$x_{TG_{VLDL}}$	Plasma VLDL-triglyceride
x_9	$x_{C_{VLDL}}$	Plasma VLDL-cholesterol
x_{10}	$x_{C_{HDL}}$	Plasma HDL-cholesterol
x_{11}	x_{FFA}	Plasma free fatty acid

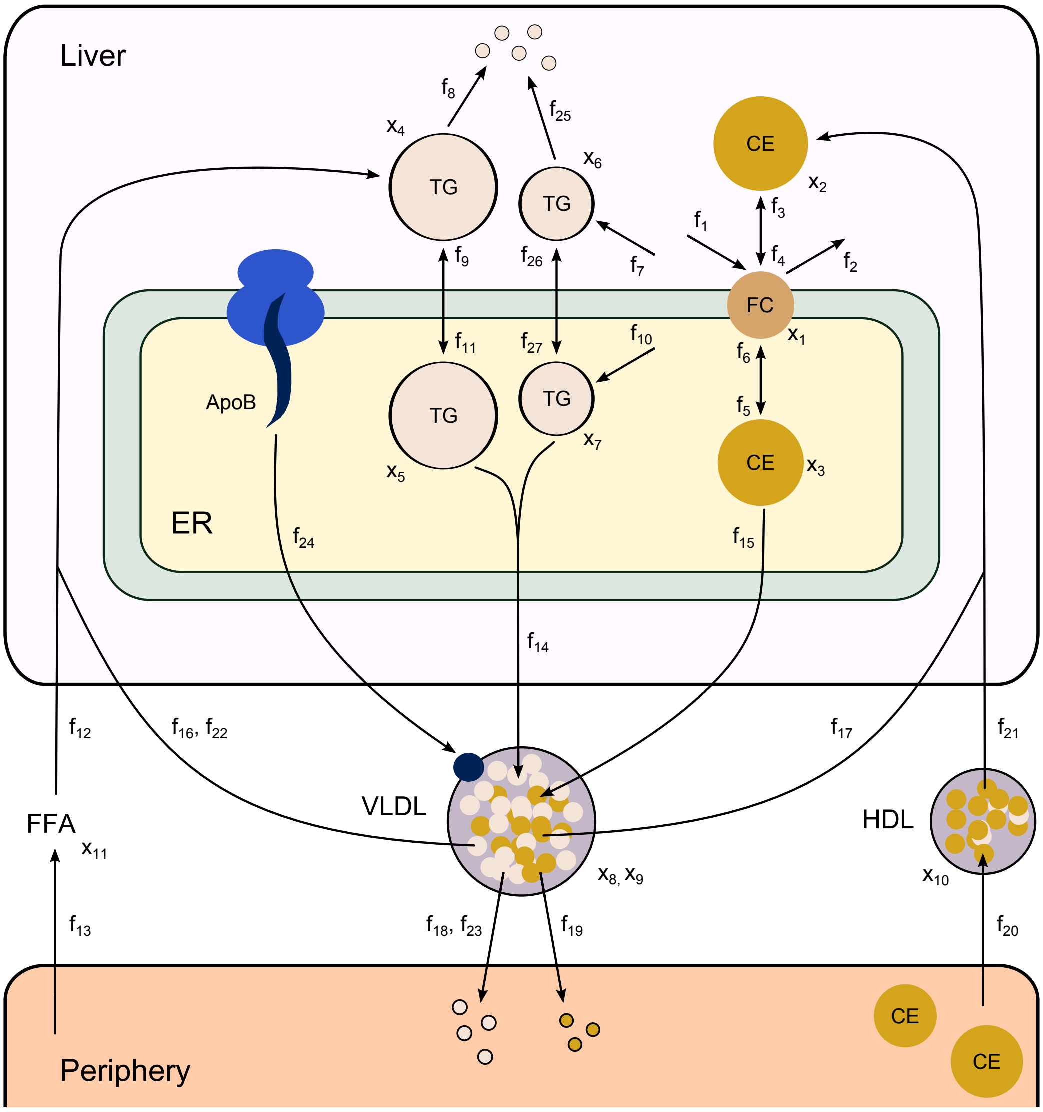

Figure S3. Computational model of hepatic lipid and plasma lipoprotein metabolism. The mathematical model is composed of three compartments representing the liver, blood plasma, and peripheral tissues. The liver compartment includes reactions comprising the production, utilization and storage of triglycerides and cholesterols, and the mobilization of these metabolites to the endoplasmic reticulum, where they are incorporated into nascent VLDL particles. The VLDL particles are secreted in the plasma compartment where they serve as nutrients for peripheral tissues. Remnant particles are taken up and cleared by the liver. The model furthermore includes the hepatic uptake of free fatty acids as well as HDL-mediated reverse cholesterol transport. ApoB, apolipoprotein B; CE, cholesterylester; ER, endoplasmic reticulum; FFA, free fatty acid; FC, free cholesterol; HDL, high-density-lipoprotein; TG, triglyceride; VLDL, very low density lipoprotein.

equations are given by:

$$
\begin{aligned}
\frac{d[x_{FC}]}{dt} &= F_{FC_{prod}} + F_{CEdef_{cyt}} + F_{CEdef_{ER}} - F_{FC_{met}} - F_{CEfor_{cyt}} - F_{CEfor_{ER}} \\
\frac{d[x_{CE_{cyt}}]}{dt} &= F_{CEfor_{cyt}} - F_{CEdef_{cyt}} + V_{plasma}\left(F_{CEupt_{hep}} + F_{CEupt_{HDL}}\right) \\
\frac{d[x_{CE_{ER}}]}{dt} &= F_{CEfor_{ER}} - F_{CEdef_{ER}} - F_{VLDL-CE} \\
\frac{d[x_{TG_{cyt}}]}{dt} &= F_{TGfor_{cyt}} - F_{TGfor_{ER}} - F_{TGmet_{cyt}} \\
&\quad + V_{plasma}\left(\frac{F_{FFA_{upt}}}{3} + F_{TGupt_{hep}} + F_{TGhyd_{hep}}\right) \\
\frac{d[x_{TG_{ER}}]}{dt} &= F_{TGfor_{ER}} - F_{TGfor_{cyt}} - F_{VLDL-TGndnl} \\
\frac{d[x_{TGdnl_{cyt}}]}{dt} &= F_{TGdnl_{cyt}} - F_{TGdnlmet_{cyt}} + F_{TGdnlfor_{cyt}} - F_{TGdnlfor_{ER}} \\
\frac{d[x_{TGdnl_{ER}}]}{dt} &= F_{TGdnl_{ER}} + F_{TGdnlfor_{ER}} - F_{TGdnlfor_{cyt}} - F_{VLDL-TGdnl} \\
\frac{d[x_{TG_{VLDL}}]}{dt} &= \frac{F_{VLDL-TG}}{V_{plasma}} - F_{TGupt_{hep}} - F_{TGupt_{per}} - F_{TGhyd_{hep}} - F_{TGhyd_{per}} \\
\frac{d[x_{C_{VLDL}}]}{dt} &= \frac{F_{VLDL-CE}}{V_{plasma}} - F_{CEupt_{hep}} - F_{CEupt_{per}} \\
\frac{d[x_{C_{HDL}}]}{dt} &= F_{CEfor_{HDL}} - F_{CEupt_{HDL}} \\
\frac{d[x_{FFA}]}{dt} &= F_{FFA_{prod}} - F_{FFA_{upt}}
\end{aligned}
$$

Where the square brackets indicate the concentration of a specific metabolite. The blood plasma volume, given by V_{plasma}, was assumed to be 1 mL [3].

Table S3. Overview and description of the fluxes included in the mathematical model.

Flux	Name	Equation	Description
f_1	F_{FCprod}	p_1	Hepatic *de novo* synthesis of free cholesterol
f_2	F_{FCmet}	$p_2[x_{FC}]$	Net hepatic catabolism of free cholesterol
f_3	$F_{CEfor_{cyt}}$	$p_3[x_{FC}]$	Hepatic synthesis of cholesteryl ester (cytosol)
f_4	$F_{CEdef_{cyt}}$	$p_4[x_{CE_{cyt}}]$	Hepatic conversion of cholesteryl ester (cytosol) to free cholesterol
f_5	$F_{CEfor_{ER}}$	$p_5[x_{FC}]$	Hepatic synthesis of cholesteryl ester (ER)
f_6	$F_{CEdef_{ER}}$	$p_6[x_{CE_{ER}}]$	Hepatic conversion of cholesteryl ester (ER) to free cholesterol
f_7	$F_{TGdnl_{cyt}}$	p_7	Hepatic *de novo* synthesis of triglyceride (cytosol)
f_8	$F_{TGmet_{cyt}}$	$p_8[x_{TG_{cyt}}]$	Hepatic catabolism of triglyceride (cytosol)
f_9	$F_{TGfor_{cyt}}$	$p_9[x_{TG_{ER}}]$	Hepatic transport of triglyceride from the ER to the cytosol
f_{10}	$F_{TGdnl_{ER}}$	p_{10}	Hepatic *de novo* synthesis of triglyceride (ER)
f_{11}	$F_{TGfor_{ER}}$	$p_{11}[x_{TG_{cyt}}]$	Hepatic transport of triglyceride from the cytosol to the ER
f_{12}	$F_{FFA_{upt}}$	$p_{12}[x_{FFA}]$	Hepatic uptake of free fatty acid
f_{13}	$F_{FFA_{prod}}$	p_{13}	Net efflux of free fatty acid from peripheral tissues to plasma
f_{14}	$F_{VLDL-TG}$	$p_{14}([x_{TG_{ER}}]+[x_{TGdnl_{ER}}])$	Hepatic secretion rate of VLDL-triglyceride
f_{15}	$F_{VLDL-CE}$	$p_{15}[x_{CE_{ER}}]$	Hepatic secretion rate of VLDL-cholesterol
f_{16}	$F_{TGupt_{hep}}$	$p_{16}[x_{TG_{VLDL}}]$	Hepatic uptake of triglyceride via whole-particle uptake
f_{17}	$F_{CEupt_{hep}}$	$p_{16}[x_{C_{VLDL}}]$	Hepatic uptake of cholesterol via whole-particle uptake
f_{18}	$F_{TGupt_{per}}$	$p_{17}[x_{TG_{VLDL}}]$	Peripheral uptake of triglyceride via whole-particle uptake
f_{19}	$F_{CEupt_{per}}$	$p_{17}[x_{C_{VLDL}}]$	Peripheral uptake of cholesterol via whole-particle uptake
f_{20}	$F_{CEfor_{HDL}}$	p_{20}	Peripheral efflux of cholesterol to HDL particles
f_{21}	$F_{CEupt_{HDL}}$	$p_{21}[x_{C_{HDL}}]$	Hepatic uptake of HDL-cholesterol
f_{22}	$F_{TGhyd_{hep}}$	$p_{18}[x_{TG_{VLDL}}]$	Hepatic uptake of triglyceride via lipolytic enzymes
f_{23}	$F_{TGhyd_{per}}$	$p_{19}[x_{TG_{VLDL}}]$	Peripheral uptake of triglyceride via lipolytic enzymes
f_{24}	$F_{apoB_{prod}}$	p_{22}	Hepatic secretion rate of apolipoprotein B
f_{25}	$F_{TGdnlmet_{cyt}}$	$p_8[x_{TGdnl_{cyt}}]$	Hepatic catabolism of *de novo* triglyceride (cytosol)
f_{26}	$F_{TGdnlfor_{cyt}}$	$p_9[x_{TGdnl_{ER}}]$	Hepatic transport of *de novo* triglyceride from the ER to the cytosol
f_{27}	$F_{TGdnlfor_{ER}}$	$p_{11}[x_{TGdnl_{cyt}}]$	Hepatic transport of *de novo* triglyceride from the cytosol to the ER
f_{28}	$F_{VLDL-TGndnl}$	$p_{14}[x_{TG_{ER}}]$	Hepatic secretion rate of non *de novo* VLDL-triglyceride
f_{29}	$F_{VLDL-TGdnl}$	$p_{14}[x_{TGdnl_{ER}}]$	Hepatic secretion rate of *de novo* VLDL-triglyceride

Calculation of the VLDL particle diameter

The following strategy was followed to calculate nascent VLDL particle diameters (D_{VLDL}). As each VLDL particle contains one apolipoprotein B particle, the number of triglyceride and cholesterylester molecules per VLDL particle can be determined by correcting the specific lipid fluxes for the number of apolipoprotein B proteins. The core volume of a VLDL particle was subsequently determined assuming a molecular volume of 946.84 mL/mol for triglyceride (TG_{mv}) and a molecular volume of 685.48 ml/mol for cholesterylester (CE_{mv}) [4]. A core radius (R_c) was calculated from the core volume assuming a spherical shape of the VLDL particles. Furthermore, the particle membrane accounts for an additional two nanometers (R_s) [5].

$$D_{VLDL} = 2\left(R_c + R_s\right) \tag{1a}$$

$$R_c = \sqrt[3]{\frac{3V_c}{4\pi}} \tag{1b}$$

$$V_c = 10^{21}\frac{TG_{cnt} \cdot TG_{mv} + CE_{cnt} \cdot CE_{mv}}{N_A} \tag{1c}$$

$$TG_{cnt} = \frac{F_{VLDL-TG}}{F_{apoB_{prod}}} \tag{1d}$$

$$CE_{cnt} = \frac{F_{VLDL-CE}}{F_{apoB_{prod}}} \tag{1e}$$

Where N_A is the constant of Avogadro.

Calculation of de novo lipogenesis

The fractional contribution of *de novo* lipogenesis was calculated as follows in the computational model:

$$FC_{DNL}(t) = \frac{[x_{TGdnl_{cyt}}](t) + [x_{TGdnl_{ER}}](t)}{[x_{TG_{cyt}}](t) + [x_{TG_{ER}}](t) + [x_{TGdnl_{cyt}}](t) + [x_{TGdnl_{ER}}](t)} \tag{2}$$

Calculation of the VLDL catabolic rate

The VLDL catabolic rate was calculated as follows in the computational model:

$$CR_{VLDL}(t) = \frac{p_{16}(t) + p_{17}(t)}{p_{16}(t_0) + p_{17}(t_0)} \tag{3}$$

References

1. Tiemann C, Vanlier J, Hilbers P, van Riel N (2011) Parameter adaptations during phenotype transitions in progressive diseases. BMC Sys Biol 5: 174.
2. Gibbons G, Islam K, Pease R (2000) Mobilisation of triacylglycerol stores. Biochim Biophys Acta 1483: 37–57.
3. Rand M (2001) Handling, restraint, and techniques of laboratory rodents. Department of Animal Care, University of Arizona .
4. Teerlink T, Scheffer P, Bakker S, Heine R (2004) Combined data from LDL composition and size measurement are compatible with a discoid particle shape. J Lipid Res 45: 954–966.
5. Miller A, Smith L (1973) Activation of lipoprotein lipase by apolipoprotein glutamic acid. J Biol Chem 248: 3359–3362.
