# Supplementary material for: Parameter Trajectory Analysis to Identify Treatment Effects of Pharmacological Interventions
Source: PLoS Comput Biol. 2013 Aug 1;9(8):e1003166. doi: 10.1371/journal.pcbi.1003166 (PMC3731221; doi:10.1371/journal.pcbi.1003166)
Supplement: Text S4 — An overview of the experimental data and corresponding spline interpolants that were used as input for ADAPT. (PDF) [file pcbi.1003166.s004.pdf]

Parameter Trajectory Analysis to Identify Treatment Effects of Pharmacological Interventions (Supporting Information Text S4)

C.A. Tiemann, J. Vanlier, M.H. Oosterveer, A.K. Groen, P.A.J. Hilbers, N.A.W. van Riel

Monte Carlo sampling of data interpolants

To enable the estimation of dynamic trajectories of metabolic parameters and fluxes, continuous dynamic descriptions of the experimental data were used as input for the computational approach. For this purpose, cubic smoothing splines were calculated that describe the dynamic trend of the experimental data. To account for experimental and biological uncertainties a collection of splines was calculated using a Monte Carlo approach. Different random samples of the experimental data were generated assuming Gaussian distributions with means and standard deviations of the data. Subsequently, for each generated sample a cubic smoothing spline was calculated. An overview of the experimental data, as well as corresponding $2D$ histograms of the splines that were used as input for ADAPT, is presented in Figure S4. A darker color represents a higher density of trajectories in that specific region and time point. The data is represented by means ± standard deviations, with an exception for the experimental data obtained via FPLC measurements. These measurements were performed on pooled mice plasma and are represented by the white dots. Measures of spread used for the Monte Carlo sampling of these quantities were estimated based on similar experiments that were performed [1]. An overview of the quantities that were experimentally observed and its relation to corresponding model components is presented in Table S4. Note that model output y_{13} was only experimentally observed for the untreated phenotype [2].

Table S4. Overview of the quantities that were measured and its relation to corresponding model components. A description of the mathematical model including an overview of the states, parameters, fluxes, and ordinary differential equations is presented in Supporting Information Text S3.

Measurement	Model output	Equation
Hepatic triglyceride	y_1	$[x_{TG_{cyt}}] + [x_{TG_{ER}}] + [x_{TGdnl_{cyt}}] + [x_{TGdnl_{ER}}]$
Hepatic cholesteryl ester	y_2	$[x_{CE_{cyt}}] + [x_{CE_{ER}}]$
Hepatic free cholesterol	y_3	$[x_{FC}]$
Plasma total cholesterol	y_4	$[x_{C_{VLDL}}] + [x_{C_{HDL}}]$
HDL-cholesterol	y_5	$[x_{C_{HDL}}]$
Plasma triglyceride	y_6	$[x_{TG_{VLDL}}]$
Plasma free fatty acid	y_7	$[x_{FFA}]$
VLDL TG/C ratio	y_8	$\frac{TG_{cnt}}{CE_{cnt}}$
VLDL diameter	y_9	D_{VLDL}
VLDL-TG production	y_{10}	$F_{VLDL-TG}$
VLDL catabolic rate	y_{11}	CR_{VLDL}
De novo lipogenesis	y_{12}	FC_{DNL}
Hepatic HDL-C uptake	y_{13}	$F_{CEupt_{HDL}}$

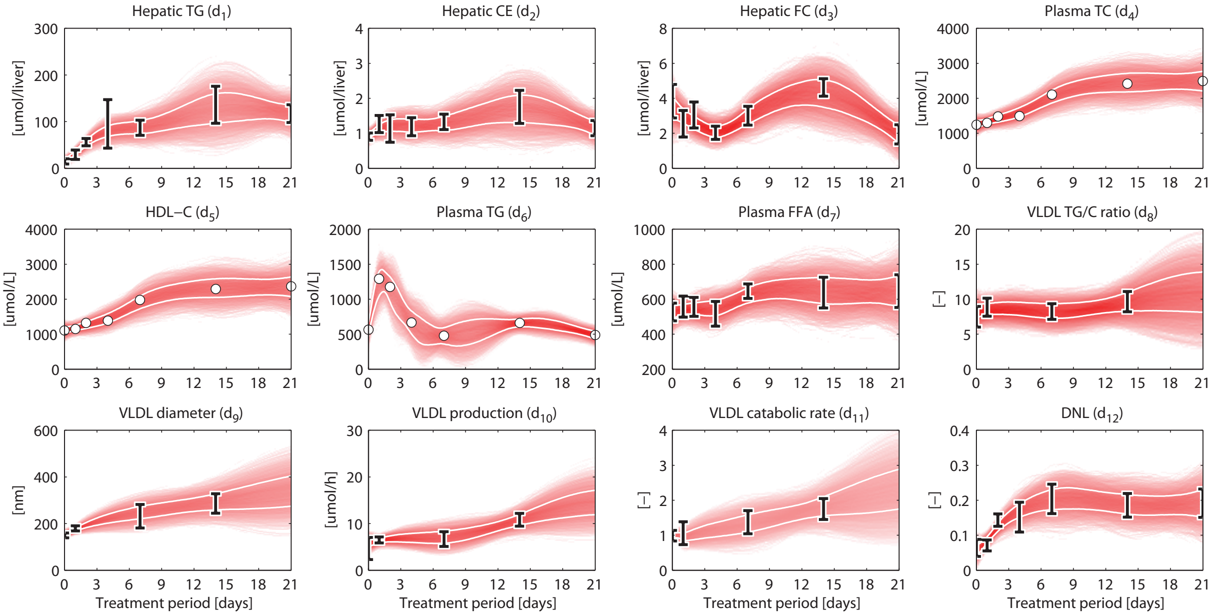

Figure S4. Experimental data and interpolants. An overview of the experimental data, as well as corresponding $2D$ histograms of the splines that were used as input for ADAPT, is presented. Data is represented by means ± standard deviations (N=5-6), with an exception for the experimental data obtained via FPLC measurements. These measurements were performed on pooled mice plasma and are represented by the white dots. The white lines enclose the central 67% of the interpolant density at each time point.

References

1. Grefhorst A, Oosterveer M, Brufau G, Boesjes M, Kuipers F, et al. (2012) Pharmacological lxr activation reduces presence of sr-b1 in liver membranes contributing to lxr-mediated induction of hdl-cholesterol. Atherosclerosis .

2. Xie C, Turley S, Dietschy J (2009) Abca1 plays no role in the centripetal movement of cholesterol from peripheral tissues to the liver and intestine in the mouse. Journal of lipid research 50: 1316–1329.
