# Supplementary material for: Parameter Trajectory Analysis to Identify Treatment Effects of Pharmacological Interventions
Source: PLoS Comput Biol. 2013 Aug 1;9(8):e1003166. doi: 10.1371/journal.pcbi.1003166 (PMC3731221; doi:10.1371/journal.pcbi.1003166)
Supplement: Text S5 — Analysis of the influence of changing the number of time steps used in ADAPT on the model outputs and regularization error. (PDF) [file pcbi.1003166.s005.pdf]

Parameter Trajectory Analysis to Identify Treatment Effects of Pharmacological Interventions (Supporting Information Text S5)

C.A. Tiemann, J. Vanlier, M.H. Oosterveer, A.K. Groen, P.A.J. Hilbers, N.A.W. van Riel

Effect of the number of time steps

An analysis was performed to investigate the influence of the number of time steps N_t, used in the estimation of parameter trajectories, on the total model error (1) and total regularization error (2):

$$\chi^2_{d_{tot}} = \sum_{n=1}^{N_t} \chi^2_d(\vec{\theta}[n])\Delta t \tag{1}$$

$$\chi^2_{r_{tot}} = \sum_{n=1}^{N_t} \chi^2_r(\vec{\theta}[n])\Delta t \tag{2}$$

To this end, ADAPT was employed for a collection of different numbers of time steps N_t, ranging from 2 to 1000 steps. For each N_t a collection of one hundred trajectories was obtained. Figure S5 presents the total model error and total regularization error as function of the number of time steps N_t. The color indicates the percentage of model outputs that describe the experimental data acceptably (green: 100% acceptable, red: 70% acceptable). A model output was considered acceptable if its value was within the 95% confidence interval of the data. The thick lines indicate the mean, whereas the scatter represents the individual error values. The total model error decreases rapidly upon increase of the number of time steps, and converges around 20 steps. Changing the number of time steps had less effect on the total regularization error, although a slightly decreasing trend can be observed converging around 200 steps. In the present study 200 time steps were used.

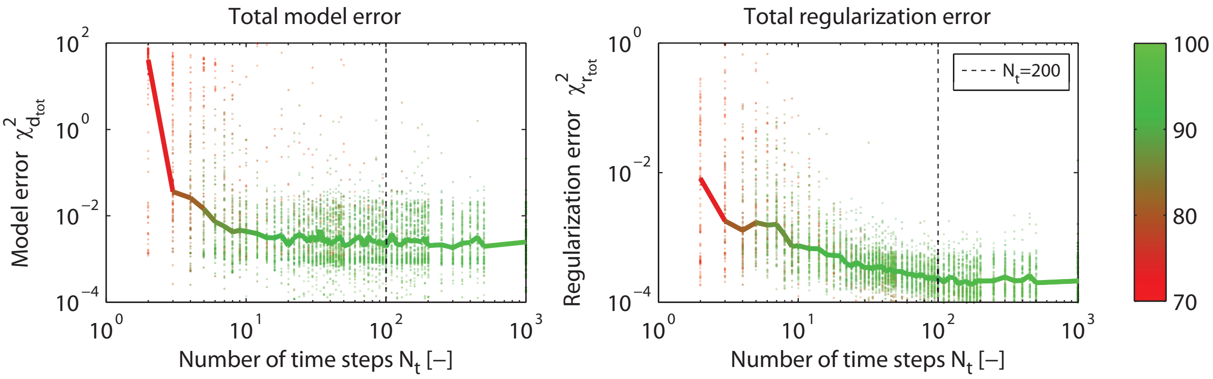

Figure S5. Effect of the number of time steps. Left) Total model error as function of the number of time steps N_t. Right) Total regularization error as function of the number of time steps N_t. The color indicates the percentage of model outputs that describe the experimental data acceptably (green: 100% acceptable, red: 70% acceptable). The thick lines indicate the mean, whereas the scatter represents the individual error values. In the present study 200 time steps were used.
