# Supplementary material for: Parameter Trajectory Analysis to Identify Treatment Effects of Pharmacological Interventions
Source: PLoS Comput Biol. 2013 Aug 1;9(8):e1003166. doi: 10.1371/journal.pcbi.1003166 (PMC3731221; doi:10.1371/journal.pcbi.1003166)
Supplement: Text S6 — Analysis of the influence of changing the regularization strength on the model outputs and regularization error. (PDF) [file pcbi.1003166.s006.pdf]

Parameter Trajectory Analysis to Identify Treatment Effects of Pharmacological Interventions (Supporting Information Text S6)

C.A. Tiemann, J. Vanlier, M.H. Oosterveer, A.K. Groen, P.A.J. Hilbers, N.A.W. van Riel

Determination of the regularization strength

It was assumed that the induced adaptations proceed progressively in time. Therefore, highly fluctuating parameter trajectories were considered to be unphysiological. To prevent the occurrence of such behavior, a regularization term, given by the sum of squared derivatives of the normalized parameter values at current step n, was included in the parameter estimation procedure. An optimized parameter set is defined as follows:

$$\hat{\vec{\theta}}[n] = \arg\min_{\vec{\theta}[n]} \left(\chi_d^2(\vec{\theta}[n]) + \lambda_r \chi_r^2(\vec{\theta}[n]) \right) \tag{1}$$

with objective functions χ_d^2 and χ_r^2 given by:

$$\chi_d^2(\vec{\theta}[n]) = \sum_{i=1}^{N_y} \left(\frac{Y_i[n] - d_i(n\Delta t)}{\sigma_i(n\Delta t)} \right)^2 \tag{2}$$

$$\chi_r^2(\vec{\theta}[n]) = \sum_{i=1}^{N_p} \left(\frac{\theta_i[n] - \theta_i[n-1]}{\Delta t} \frac{1}{\theta_i[0]} \right)^2 \tag{3}$$

with λ_r a constant determining the strength of the regularization term. A potential risk of regularization (and multi-objective optimization in general) is that for a low λ_r the regularization term has no effect, whereas for a large λ_r the parameter estimation algorithm might minimize the regularization term while describing the experimental data inaccurately. To determine a suitable value for λ_r, ADAPT was employed for a large set of different λ_r constants. For each λ_r a collection of one hundred trajectories was obtained. Subsequently, the effect of λ_r on the total model error (4) and the total regularization error (5) was investigated:

$$\chi_{d_{tot}}^2 = \sum_{n=1}^{N_t} \chi_d^2(\vec{\theta}[n]) \Delta t \tag{4}$$

$$\chi_{r_{tot}}^2 = \sum_{n=1}^{N_t} \chi_r^2(\vec{\theta}[n]) \Delta t \tag{5}$$

In this specific study all optimizations were performed with an identical set of data interpolants. Furthermore, for each series of λ_r constants, identical initial conditions (states and parameters) were used for the optimizations. Hence, in this way observed differences can directly be attributed to changes in λ_r. Figure S6 presents the total model error and total regularization error as function of λ_r. Here, the color indicates the percentage of model outputs that describe the experimental data acceptably (green: 100% acceptable, red: 0% acceptable). A model output was considered acceptable if its value was within the 95% confidence interval of the data. A well-defined switching point can be observed (around $\lambda_r = 10$) where the regularization term becomes dominant and the mathematical model in not able to describe the experimental data accurately any more. Note that a small λ_r is already sufficient to minimize parameter changes and fluctuations, while the experimental data is still described accurately. It is preferred to bias the data fitting as little as possible and therefore in further studies a λ_r of 0.01 was selected.

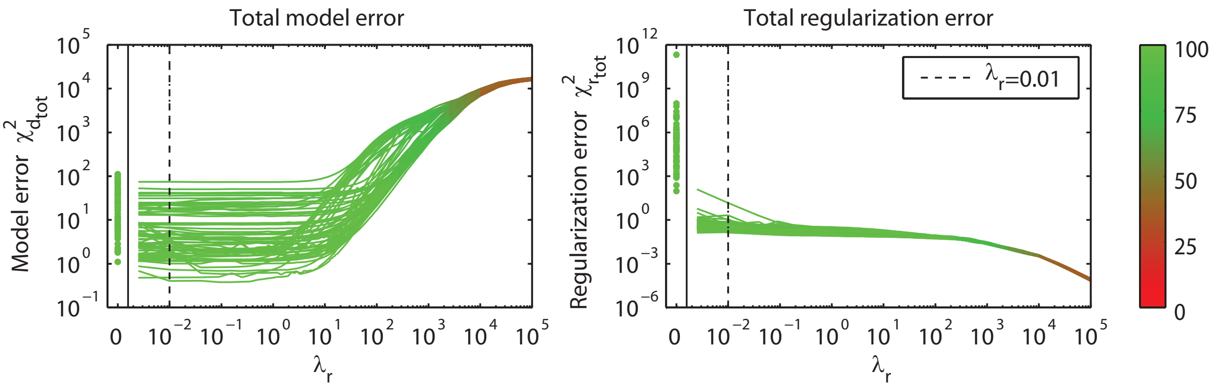

Figure S6. Effect of regularization strength. Left) Total model error as function of λ_r. Right) Total regularization error as function of λ_r. The color indicates the percentage of model outputs that describe the experimental data acceptably (green: 100% acceptable, red: 0% acceptable). Note that a small λ_r is already sufficient to minimize parameter changes and fluctuations, while describing the experimental data still accurately. For further studies a λ_r of 0.01 was selected (dashed line).
