# Supplementary material for: Parameter Trajectory Analysis to Identify Treatment Effects of Pharmacological Interventions
Source: PLoS Comput Biol. 2013 Aug 1;9(8):e1003166. doi: 10.1371/journal.pcbi.1003166 (PMC3731221; doi:10.1371/journal.pcbi.1003166)
Supplement: Text S7 — Analysis of unwanted side effects when targeting or to prevent the excessive accumulation of triglycerides in the liver upon T0901317 treatment. (PDF) [file pcbi.1003166.s007.pdf]

Parameter Trajectory Analysis to Identify Treatment Effects of Pharmacological Interventions (Supporting Information Text S7)

C.A. Tiemann, J. Vanlier, M.H. Oosterveer, A.K. Groen, P.A.J. Hilbers, N.A.W. van Riel

Analysis and targeting of unwanted side effects

Besides its beneficial effects on cholesterol metabolism, pharmacological LXR activation also induces unwanted side effects such as the accumulation of triglycerides in the liver. The processes for which the hepatic triglyceride level is sensitive are potential targets for future interventions to prevent the unwanted side effect of abnormal triglyceride accumulation in the liver. To illustrate this, we performed a computational analysis to investigate whether it is possible to prevent hepatic triglyceride accumulation upon T0901317 treatment by targeting one of the sensitive quantities, i.e., the triglyceride catabolism capacity (p_8). The 10000 parameter trajectory sets obtained from the previous analysis were used as input to simulate the computational model, with an exception for p_8. This parameter is iteratively re-estimated (while keeping the other parameters fixed according to their trajectories) to maintain a constant hepatic triglyceride level ($x_4 + x_5 + x_6 + x_7$) during the treatment intervention. Re-estimation of parameter p_8, while forcing the total hepatic triglyceride pool to remain constant in time, indicates that this objective could be achieved by designing an intervention that maintains the triglyceride catabolism capacity of untreated mice. Furthermore, applying this perturbation induced only negligible adaptations in the other metabolite concentrations (Figure S7, left versus middle column). Another option could be to target the triglyceride transport (f_{11} and p_{11}). Indeed, it is possible to prevent the hepatic triglyceride accumulation by targeting p_{11}. However, this induces another unwanted side effect, that is the accumulation of triglycerides in the plasma (Figure S7, right column).

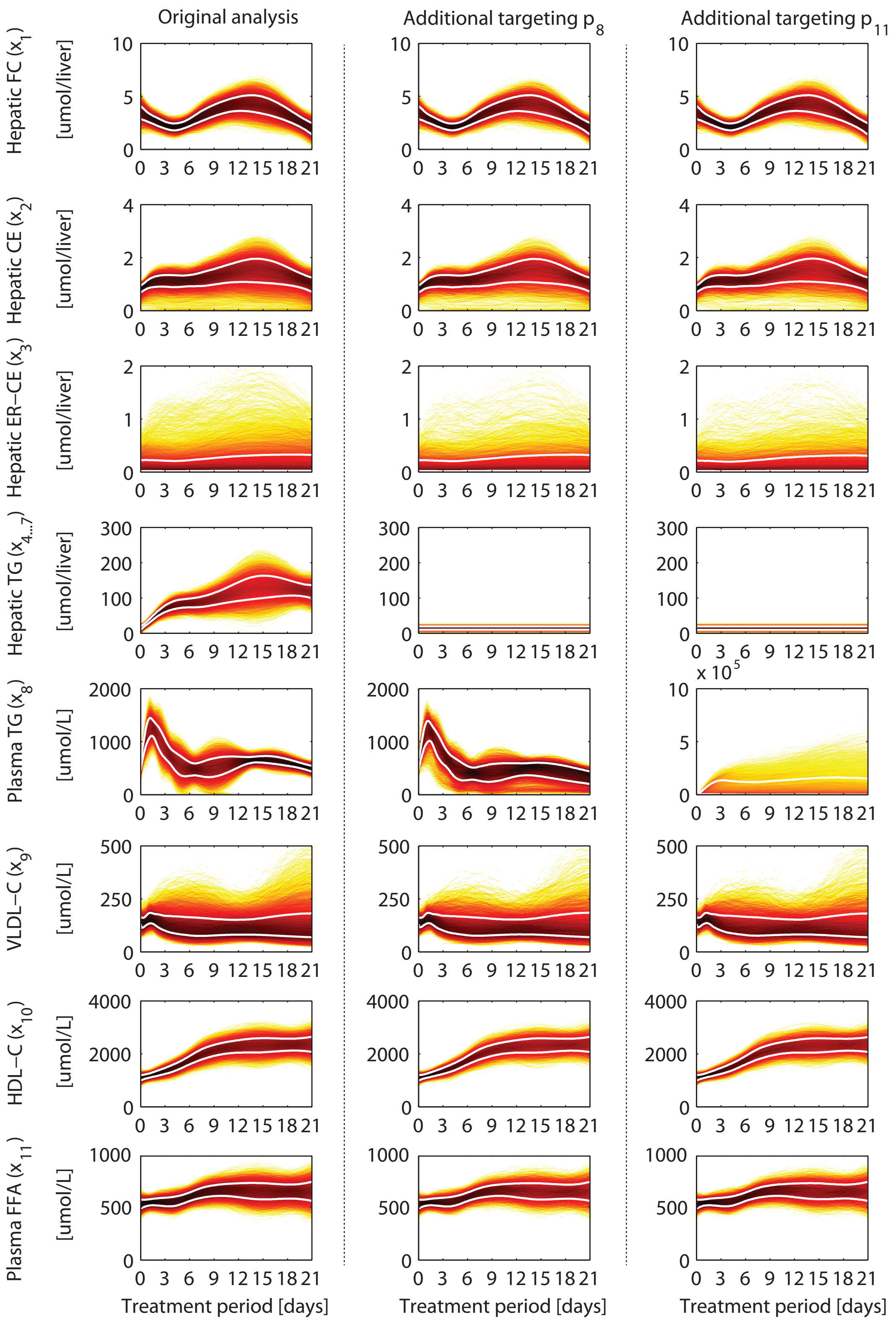

Figure S7. Targeting of unwanted side effects. A computational analysis was performed to investigate whether it is possible to maintain normal hepatic triglyceride levels upon T0901317 treatment by targeting the triglyceride catabolism capacity p_8 (middle column) or the triglyceride transport capacity p_{11} (right column). This is indeed the case for both targeting strategies. For p_8 only negligible differences in the other metabolites can be observed compared to the original analysis (left column). However, targeting p_{11} could result in considerable accumulation of triglycerides in the plasma. The white lines enclose the central 67% of the densities.
