# Supplementary material for: Parameter Trajectory Analysis to Identify Treatment Effects of Pharmacological Interventions
Source: PLoS Comput Biol. 2013 Aug 1;9(8):e1003166. doi: 10.1371/journal.pcbi.1003166 (PMC3731221; doi:10.1371/journal.pcbi.1003166)
Supplement: Text S8 — Analysis of relative adaptations of trajectories to identify consistent model behavior. (PDF) [file pcbi.1003166.s008.pdf]

Parameter Trajectory Analysis to Identify Treatment Effects of Pharmacological Interventions (Supporting Information Text S8)

C.A. Tiemann, J. Vanlier, M.H. Oosterveer, A.K. Groen, P.A.J. Hilbers, N.A.W. van Riel

Relative adaptations of trajectories

Several parameter trajectories (or trajectories of states and fluxes) are not well-constrained by the experimental data (see also Supporting Information Text S9). For these model quantities it is difficult (or impossible) to conclude whether and how they are affected by the treatment intervention. However, despite the large uncertainty, some trajectories display quite consistent behavior with respect to corresponding values of the untreated phenotype. Hence, exploring relative changes of trajectories with respect to the untreated phenotype could provide useful information about how the quantity is affected by the treatment. An example is illustrated in Figure S8. The top row displays several flux trajectories associated with hepatic cholesterol metabolism that are not well-constrained. The bottom row displays the relative changes of the these fluxes, which provides a more clear picture of how these fluxes behave in time.

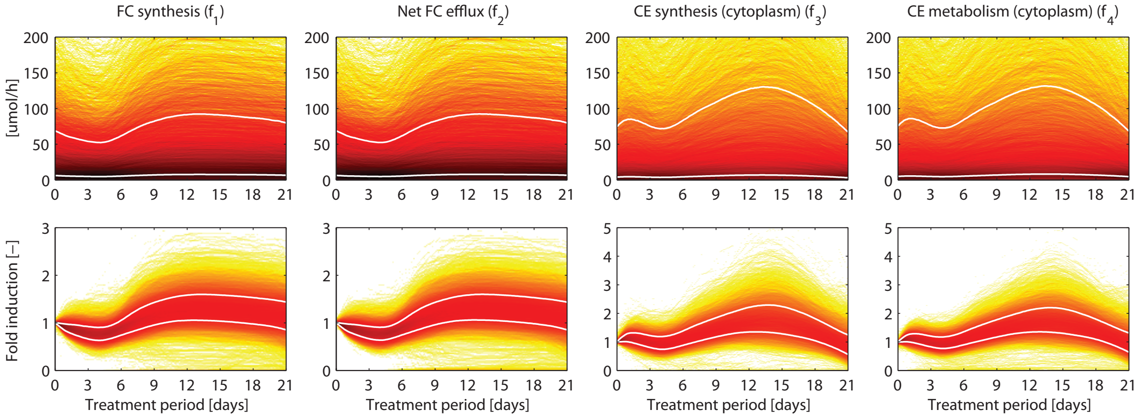

Figure S8. Relative adaptation of trajectories. The top row displays several flux trajectories associated with hepatic cholesterol metabolism that are not well-constrained. The bottom row displays the relative changes of the these fluxes, which provides a more clear picture of how these fluxes behave in time. Hence, despite the large uncertainty, some trajectories display quite consistent behavior with respect to corresponding values of the untreated phenotype.
