# Supplementary material for: Parameter Trajectory Analysis to Identify Treatment Effects of Pharmacological Interventions
Source: PLoS Comput Biol. 2013 Aug 1;9(8):e1003166. doi: 10.1371/journal.pcbi.1003166 (PMC3731221; doi:10.1371/journal.pcbi.1003166)
Supplement: Text S9 — Analysis of the identifiability of parameters for the untreated phenotype, using ADAPT and the Profile Likelihood method. (PDF) [file pcbi.1003166.s009.pdf]

Parameter Trajectory Analysis to Identify Treatment Effects of Pharmacological Interventions (Supporting Information Text S9)

C.A. Tiemann, J. Vanlier, M.H. Oosterveer, A.K. Groen, P.A.J. Hilbers, N.A.W. van Riel

Parameter profile likelihood of the untreated phenotype

To account for variations in the dynamic behavior as well as experimental and biological uncertainties, a collection of smoothing splines was calculated using a Monte Carlo approach in which random samples of the experimental data were generated. This provides the possibility to determine how data uncertainty is propagated through model parameters, and hence allows to distinguish between parameters that are well-constrained and those that display a large spread of possible outcomes. A commonly used approach to assess the identifiability of parameters is the Profile Likelihood method [1,2]. In this method parameter profiles are obtained as follows. A parameter i is selected and corresponding profile is initiated at the best fit parameters. Parameter i is subsequently changed, after which all other parameters are re-optimized. This process is subsequently repeated until the fit becomes unacceptable. The likelihood ratio test can be used to determine whether a fit is statistically acceptable for a given significance level [2]. Here, the Profile Likelihood analysis is performed for parameters corresponding to the untreated phenotype for which the objective function is given by:

$$\chi_0^2(\vec{\theta}) = \sum_{i=1}^{N_y} \left(\frac{y_{ss,i}(\vec{\theta}) - d_i(0)}{\sigma_i(0)} \right)^2 \quad (1)$$

The sum of squared differences along the profile can be written as:

$$\chi_{PL,i}^2(c) = \min_{\theta_{j \neq i}} \left[\chi_0^2(\vec{\theta} | \theta_i = c) \right] \quad (2)$$

The top row of Figure S9 shows the profiles of several parameters. The dashed line indicates the threshold corresponding to a significance level of 0.33. Examples of parameters have been depicted that are either identifiable (p_{20} to p_{22}), practical unidentifiable (p_{17}), or structural unidentifiable (p_9). The second row displays the parameter distributions obtained with the Monte Carlo approach employed in ADAPT. These distributions are agree well with the results obtained from the Profile Likelihood analysis. Note that the (un)certainty of the parameters of the untreated phenotype is also reflected in corresponding trajectories during the treatment intervention (Figure S9, bottom row). A challenge remains to apply the Profile Likelihood method (or other identifiability / uncertainty analysis methods) on parameter trajectories. One problem is to define (independently from ADAPT) reference parameter values for the initial ($n = 0$) and previous step ($n - 1$) which are used in the regularization objective function $\chi_r^2(\vec{\theta}[n])$. Another complication is that the Profile Likelihood method becomes computational intractable, because each profile likelihood step requires all other parameters of the full treatment period to be re-estimated. For the present case this would concern $(N_p - 1) * N_t = 4200$ parameters.

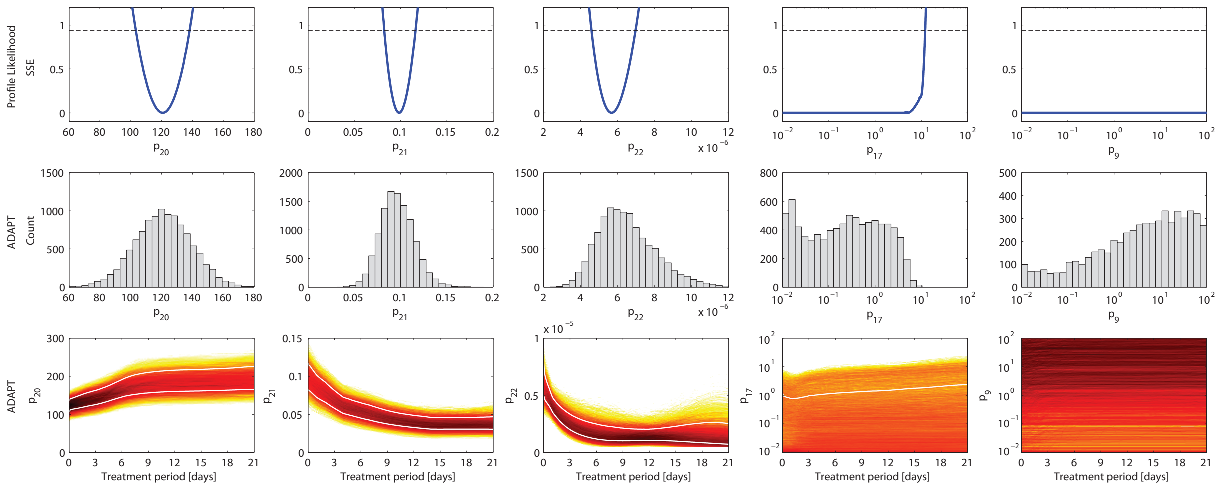

Figure S9. Identifiability of parameters. Top row) Profile likelihood of several parameters of the untreated phenotype. The dashed line indicates the threshold corresponding to a significance level of 0.33. Middle row) Parameter distributions for the untreated phenotype obtained with the Monte Carlo approach employed in ADAPT. Bottom row) Trajectories of corresponding parameters.

References

1. Vanlier J, Tiemann C, Hilbers P, van Riel N (2012) An integrated strategy for prediction uncertainty analysis. Bioinformatics 28: 1130–1135.

2. Raue A, Kreutz C, Maiwald T, Bachmann J, Schilling M, et al. (2009) Structural and practical identifiability analysis of partially observed dynamical models by exploiting the profile likelihood. Bioinformatics 25: 1923.
