# Supplementary material for: Parameter Trajectory Analysis to Identify Treatment Effects of Pharmacological Interventions
Source: PLoS Comput Biol. 2013 Aug 1;9(8):e1003166. doi: 10.1371/journal.pcbi.1003166 (PMC3731221; doi:10.1371/journal.pcbi.1003166)
Supplement: Text S10 — Comparison of model outputs obtained with time-dependent parameters used in ADAPT and an analysis using time-constant parameters. (PDF) [file pcbi.1003166.s010.pdf]

Parameter Trajectory Analysis to Identify Treatment Effects of Pharmacological Interventions (Supporting Information Text S10)

C.A. Tiemann, J. Vanlier, M.H. Oosterveer, A.K. Groen, P.A.J. Hilbers, N.A.W. van Riel

Time-dependent versus time-constant parameters

The computational workflow of ADAPT was carried out using the computational model and the acquired experimental data. Parameter trajectories were estimated using 200 time steps. A small value of 0.01 for regularization factor λ_r was chosen to bias the data fitting as little as possible. A collection of 10000 acceptable parameter trajectory sets was obtained describing the experimental data. Figure S10 shows the model outputs obtained with ADAPT. These results are in very good agreement with the acquired experimental data (and corresponding data interpolants presented in Figure S12).

A question that could arise is whether the time-dependent adaptations in model parameters are really required to describe the experimental data. One would like to test this by performing a similar analysis with time-constant parameters. However, note that the computational model does not include any mathematical descriptions of processes involved in LXR activation and its transcriptional response. Hence, it is not possible to mimic the treatment intervention by increasing the activity of LXR, simply because it is not modeled. Performing a simulation with time-constant parameters would result in a continuation of the steady-state system output of the untreated phenotype.

To establish whether the parameters have to change during the treatment intervention to describe the experimental data, it was investigated whether the data could be described by inducing a step-wise response in the model parameters at $t = 0$ (for $t > 0$ the parameters remain constant in time). For this purpose the following two-step parameter estimation protocol was applied. First, model parameters were estimated to describe the experimental data of the untreated phenotype:

$$\hat{\vec{\theta}}_0 = \arg\min_{\vec{\theta}} \chi_0^2(\vec{\theta}) \tag{1}$$

$$\chi_0^2(\vec{\theta}) = \sum_{i=1}^{N_y} \left(\frac{y_{ss,i}(\vec{\theta}) - d_i(0)}{\sigma_i(0)} \right)^2 \tag{2}$$

where N_y is the number of measurement signals, $\vec{y}_{ss}$ the steady-state model outputs, $\vec{d}$ the means of the experimental data of the untreated phenotype and $\vec{\sigma}$ corresponding standard deviations. Subsequently, step-wise changes $\vec{\delta\theta}$ with respect to the initial parameter set $\hat{\vec{\theta}}_0$ were estimated to minimize the difference between the model outputs and corresponding experimental data at different stages of the treatment:

$$\hat{\vec{\delta\theta}} = \arg\min_{\vec{\delta\theta}} \chi^2(\vec{\delta\theta}) \tag{3}$$

$$\chi^2(\vec{\delta\theta}) = \sum_{i=1}^{N_y} \sum_{j=1}^{N_{t_i}} \left(\frac{y_i(t_j, \hat{\vec{\theta}}_0 + \vec{\delta\theta}) - d_i(t_j)}{\sigma_i(t_j)} \right)^2 \tag{4}$$

where N_{t_i} represents the number of time points for data series d_i. Note that for the present case no spline interpolants of the experimental data were used in the parameter estimation. This was not required as the model parameters were not re-estimated iteratively. The optimization procedure was repeated a thousand times. The results are depicted in Figure S11. Clearly, a step-wise response in the parameters is not sufficient to describe the experimental data.

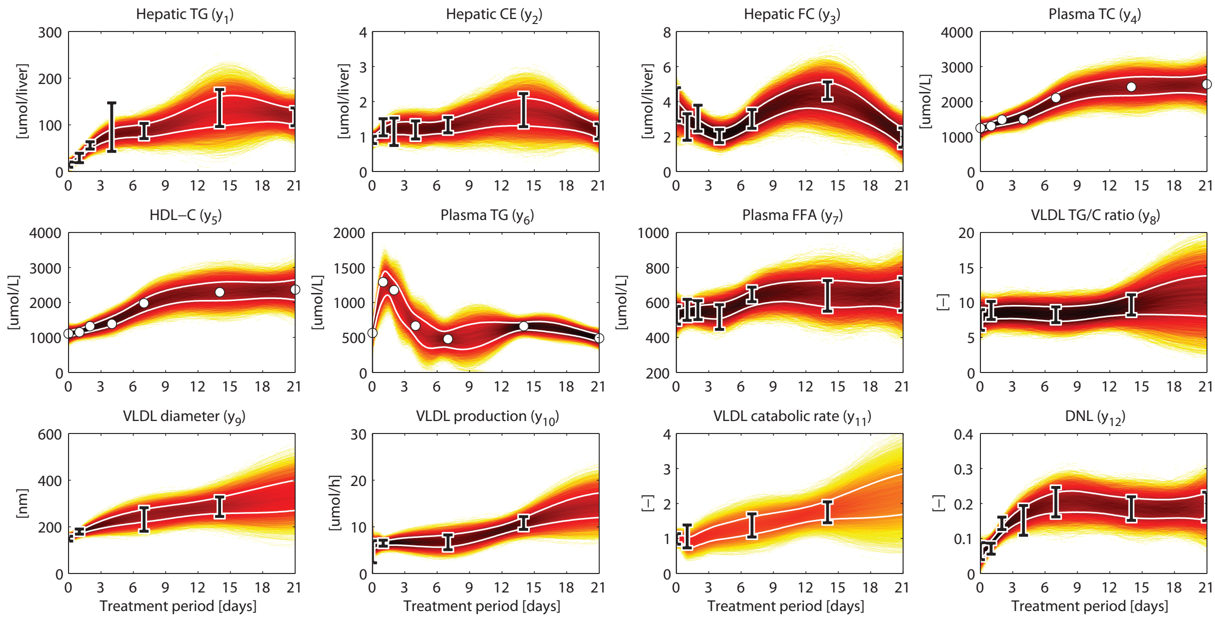

Figure S10. Model outputs obtained with ADAPT. 2D histograms were calculated from the 10000 acceptable sets to determine the density of trajectories during the treatment period. A darker color represents a higher density of trajectories in that specific region and time point. The white lines enclose the central 67% of the densities. The results are in very good agreement with the acquired experimental data (and corresponding data interpolants presented in Figure S12).

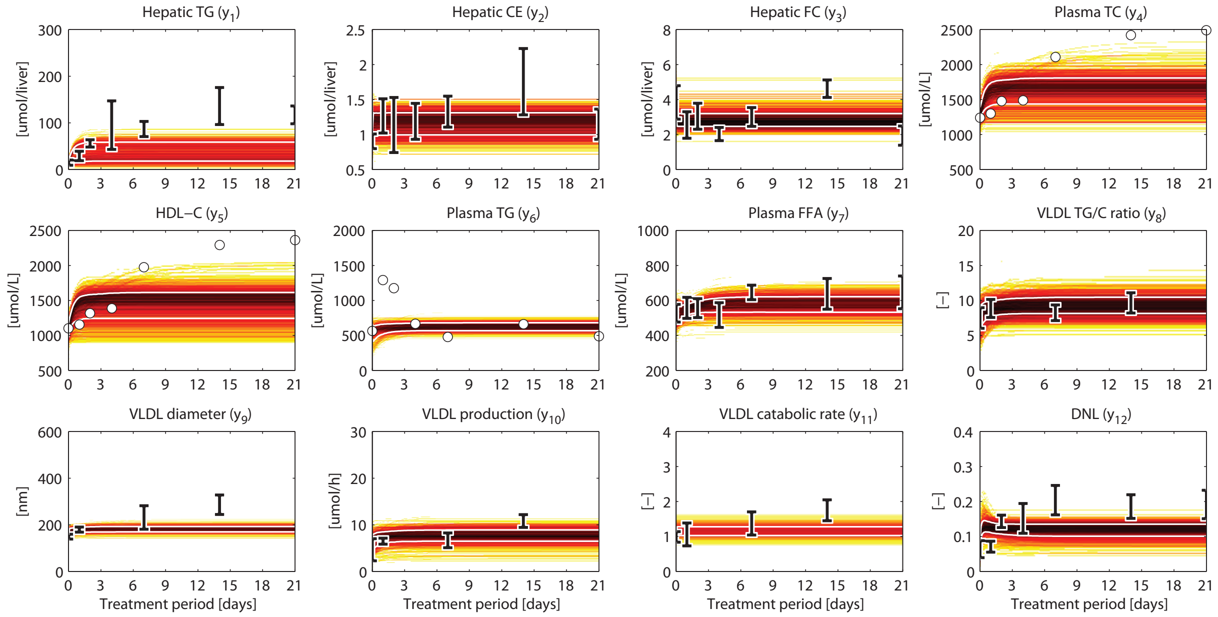

Figure S11. Model outputs obtained with a step-wise response in the parameters. 2*D* histograms were calculated from 1000 sets to determine the density of model outputs during the treatment period. A darker color represents a higher density of trajectories in that specific region and time point. The white lines enclose the central 67% of the densities. Clearly, a step-wise response in the parameters is not sufficient to describe the experimental data.

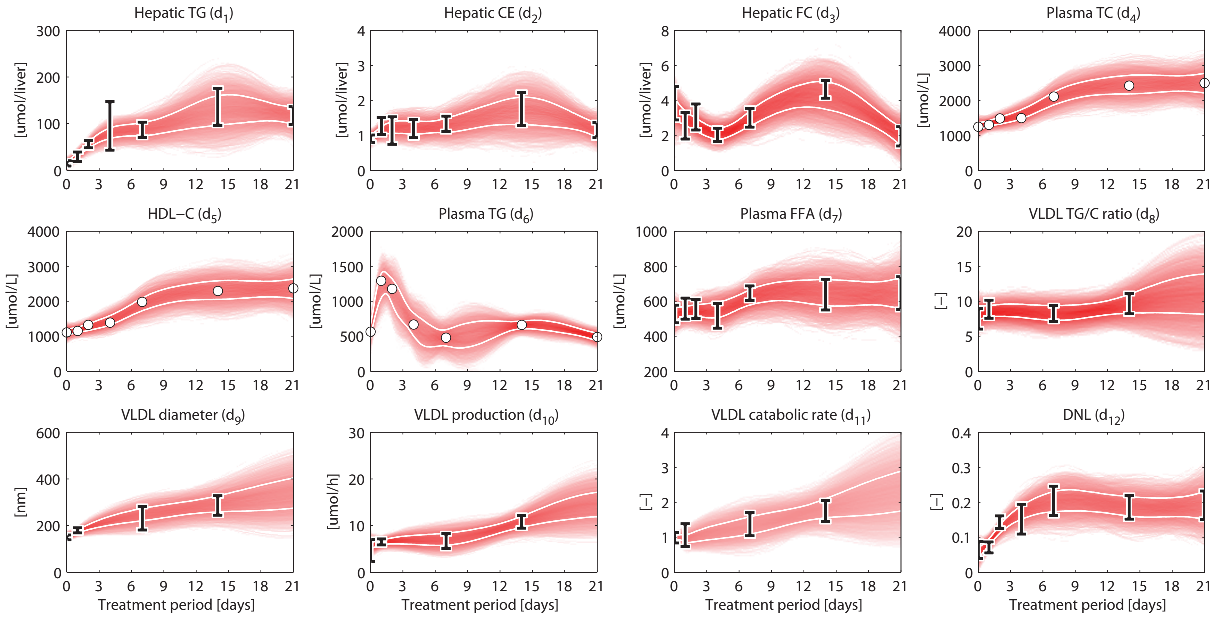

Figure S12. Experimental data and interpolants. An overview of the experimental data, as well as corresponding $2D$ histograms of the splines that were used as input for ADAPT, is presented. Data is represented by means ± standard deviations (N=5-6), with an exception for the experimental data obtained via FPLC measurements. These measurements were performed on pooled mice plasma and are represented by the white dots. The white lines enclose the central 67% of the interpolant density at each time point.
